# Supplementary material for: Timing of gene expression in a cell‐fate decision system
Source: Mol Syst Biol. 2018 Apr 25;14(4):e8024. doi: 10.15252/msb.20178024 (PMC5916086; doi:10.15252/msb.20178024)
Supplement: Supplementary file 1 — Appendix [file MSB-14-e8024-s001.pdf]

Appendix

Timing of gene expression in a cell-fate decision system

Delphine Aymoz[1], Carme Solé[2], Jean-Jerrold Pierre[1], Marta Schmitt[1], Eulàlia de Nadal[2], Francesc Posas[2], Serge Pelet[1*]
[1] Department of Fundamental Microbiology, University of Lausanne, Lausanne, Switzerland
[2] Cell Signaling Research Group, Departament de Ciències Experimentals i de la Salut Universitat Pompeu Fabra, Barcelona Spain

correspondence to: serge.pelet@unil.ch

This PDF file includes:

Appendix Figures S1 to S21
Appendix Tables S1 to S4

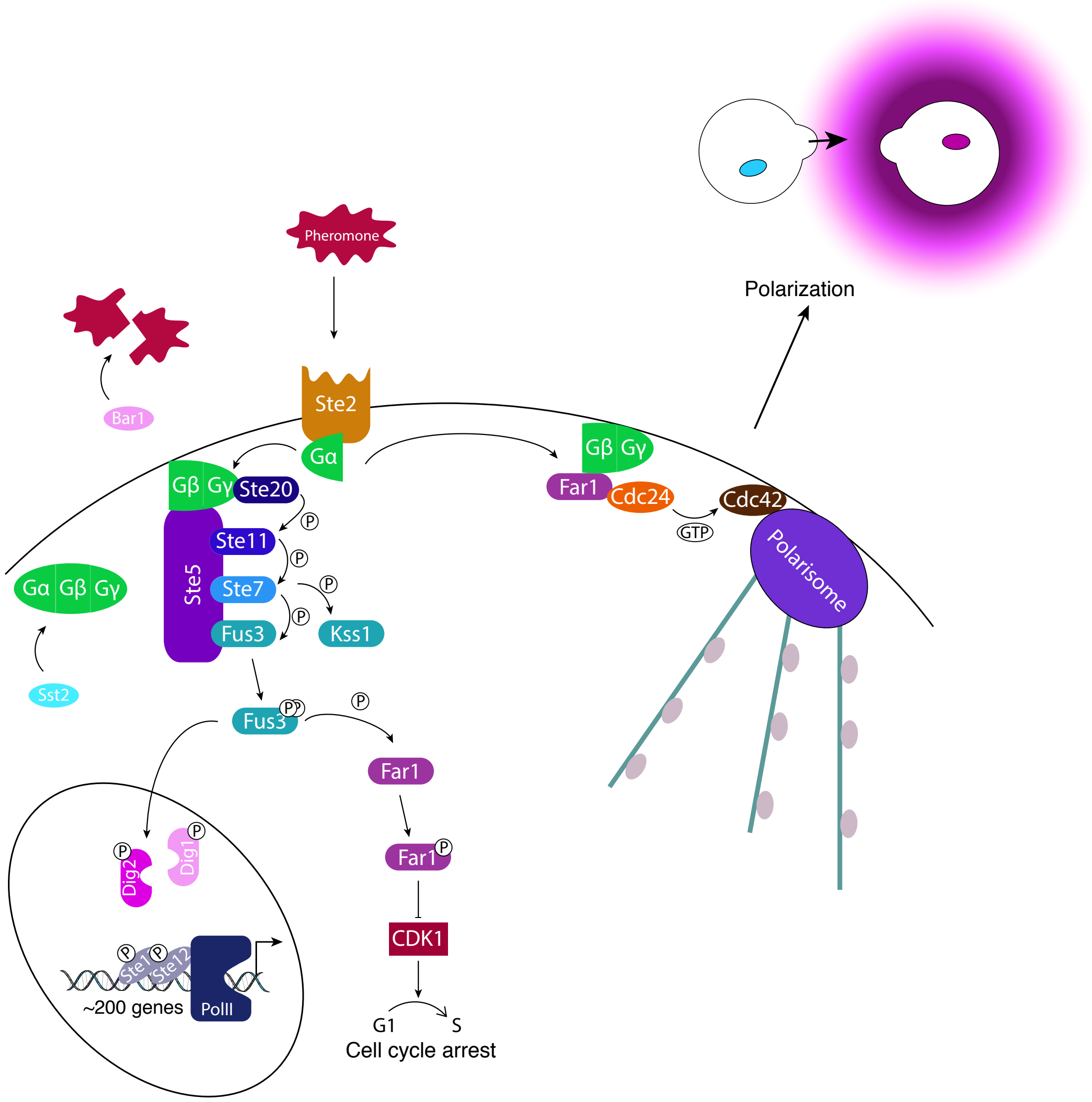

Appendix Figure S1

Schematic of the mating pathway in *S. cerevisiae*.

Presence of pheromone is detected by the G-protein coupled receptor Ste2, leading to the activation of the MAPK pathway composed of Ste11, Ste7 and Fus3 that all interact with the scaffold protein Ste5. The MAPK Kss1 is activated in parallel to Fus3 but plays a less important role in this process. MAPK activity will induce the expression of more than 200 genes through de-repression of Ste12. In parallel, phosphorylation of Far1 will inhibit cell-cycle progression and allow the proper orientation of the mating projection towards the pheromone gradient and the mating partner.

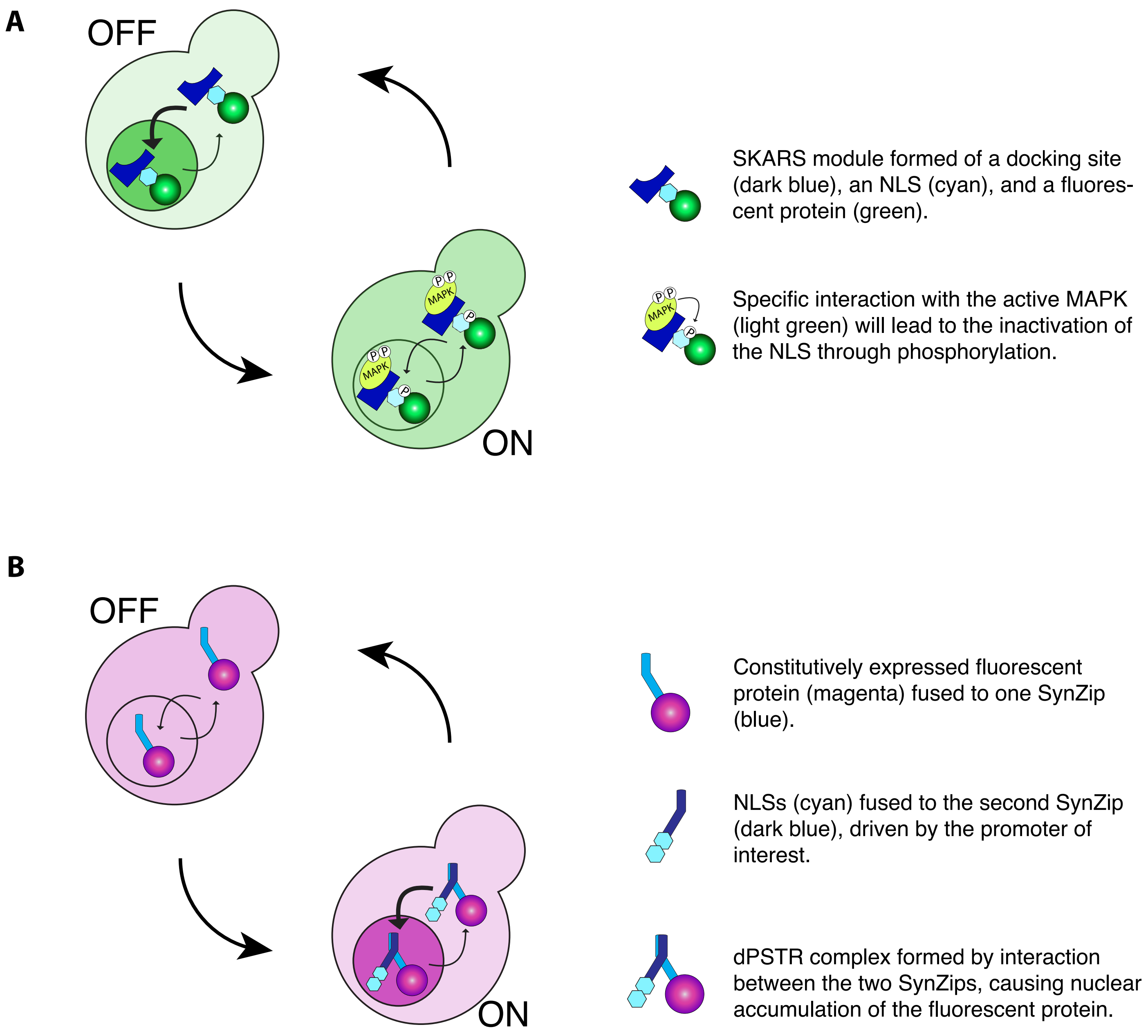

Appendix Figure S2

Principle of the relocation sensors used for the study.

A. Principle of the Synthetic Kinase Activity Relocation Sensor (SKARS, Durandau *et al.,* BMC Biology, 2015). The SKARS is formed by a docking site that can specifically interact with a kinase of interest, a phosphorylatable NLS, and a fluorescent protein for visualization. When the kinase of interest is inactive, the NLS is functional and the fluorescent signal is nuclear. Phosphorylation of the NLS by the active kinase will lower its efficiency, inducing the relocation of the fluorescent signal throughout the cell.

B. Schematic representation of the dynamic Protein Synthesis Translocation Reporter (dPSTR, Aymoz *et al.*, Nat Commun, 2016). The dPSTR converts protein expressoin arising from a promoter of interest into a relocation of a constitutive fluorescent signal from the cytoplasm into the nucleus of cells. It is based on two transcriptional units carried on a sole uniquely integrated vector. The fluorescent protein in constitutively expressed and present all over the cell in the dPSTR OFF state. A second peptide driven by the promoter of interest carries two Nuclear Localization Signals (NLS), allowing the nuclear recruitment of any protein. The physical interaction between the fluorescent protein and the induced peptide is taking place through the interaction of small synthetic peptides called SynZips, providing strong and specific interaction. The whole complex is then recruited in the nucleus, changing the fluorescent signal arising from the cytoplasm and the nucleus.

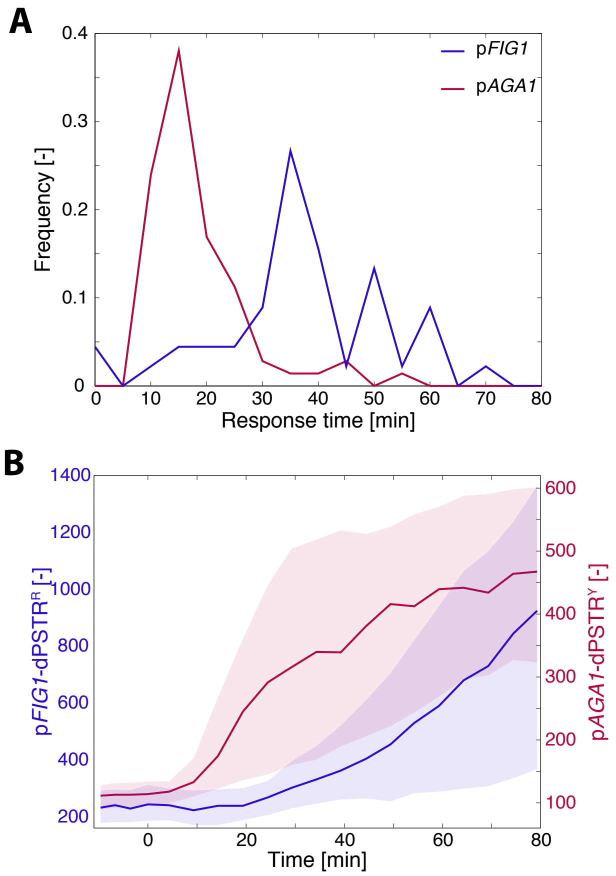

Appendix Figure S3

Expression response of signaling competent cells

A. Distribution of the response time for the indicated dPSTR for cells activating the kinase within the 10min following the stimulation. These data are extracted from the same experiments used in panels C and D of Figure 1.

B. Population median (solid line) of the nuclear enrichment of the red p*FIG1*-dPSTR^{R} (left axis, blue) or the p*AGA1*-dPSTR^{Y} (right axis, red) for the strain used in panels E to G of Figure 1. Shaded areas represent the 25-75 percentiles of the single cell responses.

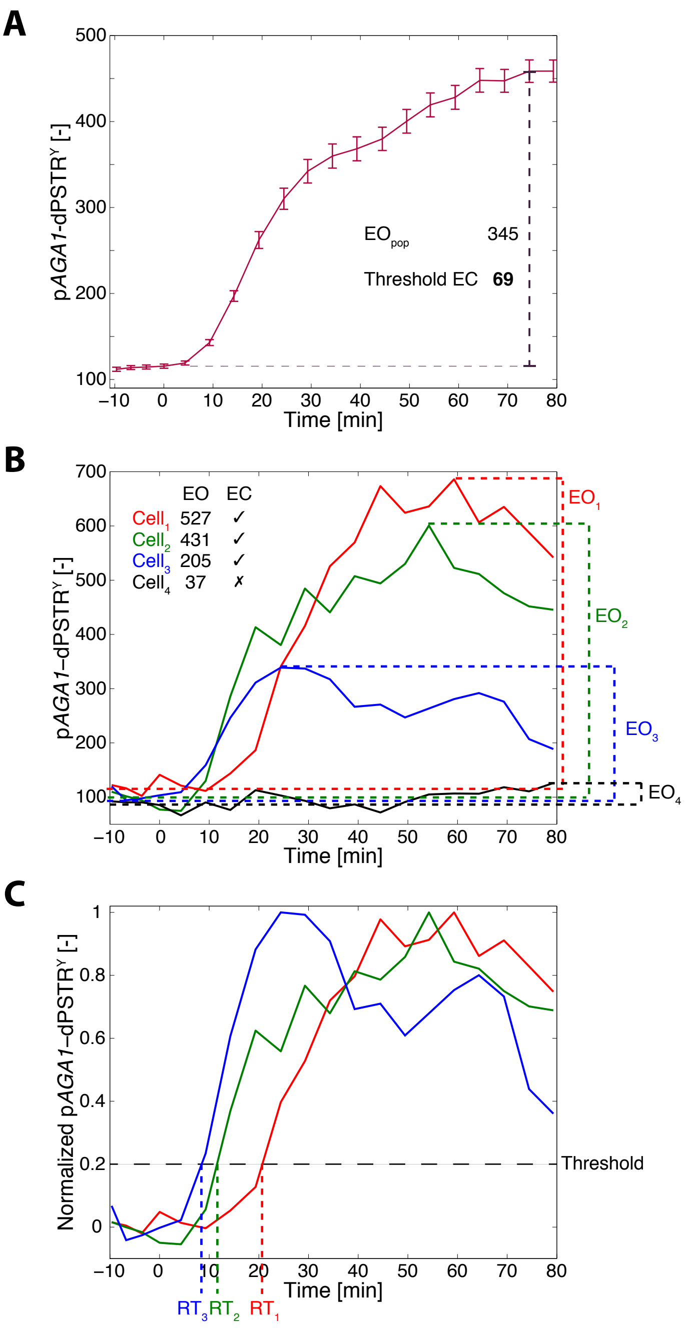

Appendix Figure S4

Metrics calculation

A. Population average nuclear enrichment of the p*AGA1*-dPSTR^{Y} in course of time. Nuclear enrichment is the difference between nuclear and cytoplasmic fluorescence. All cells of the sample that passed quality control are taken in account here (low variability on nuclear and cell area and in nuclear CFP fluorescence). Error bars represent the SEM. The expression output (EO) of the population (EO_{pop}) is calculated from the average of all smoothed traces as the difference between the maximum of the trace and the mean of the three first time points before stimulation. The threshold for expressing cells (EC) is arbitrarily set at 20% of EO_{pop}.

B. Nuclear enrichment of the p*AGA1*-dPSTR^{Y} in three single cells in course of time, before smoothing of the traces. EO of each single cell is calculated as the difference between the maximum of the single cell smoothed trace and the mean of the three first time points, as illustrated by the doted lines on the right of the plot. An expressing cell (EC) is a cell that overcomes the threshold defined arbitrarily as 20% of the EO_{pop}.

C. Normalized traces of expressing single cells are used to determine the response time (RT). All expressing cells are normalized between 0 and 1. The response time is determined as the first time point exceeding 0.2, meaning 20% of their own expression.

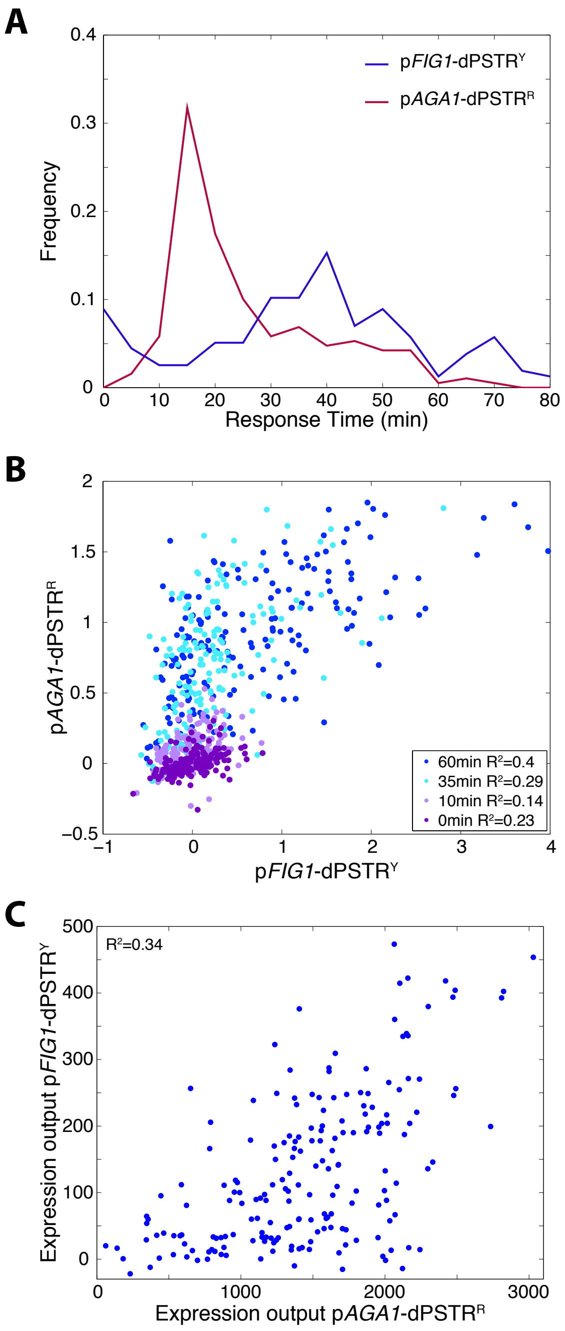

Appendix Figure S5

Consistent differential kinetics between promoters after inverting the dPSTRs FP and SynZip pairs

A. Histograms of the response time of p*FIG1*-dPSTR^Y and p*AGA1*-dPSTR^R in the same strain (Total number of cells: 193. Number of expressing cells considered in this histograms: 157 for p*FIG1*-dPSTR^Y and 189 for p*AGA1*-dPSTR^R). Note the similarity with Figure 1F.

B. Instant correlation of nuclear enrichment of the two dPSTRs at time 0, 10, 25 and 60min after stimulation by pheromone. Note that p*AGA1*-dPSTR^R is induced before p*FIG1*-dPSTR^Y.

C. Correlation of the expression output of the two dPSTRs for all the cells of the experiment.

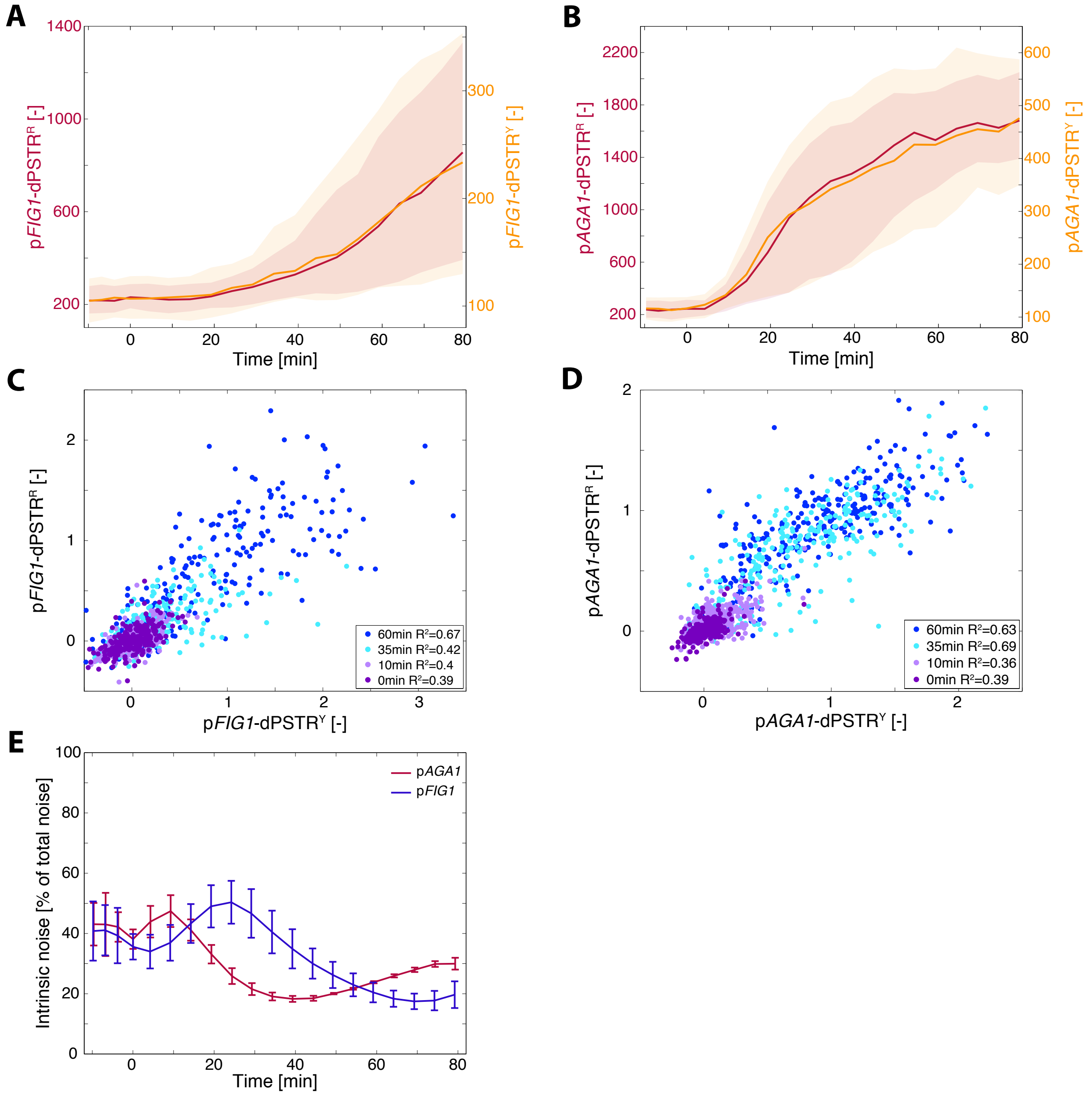

Appendix Figure S6

Temporal evolution of noise in p*FIG1* and p*AGA1* induction

A. and B. Nuclear enrichment of p*FIG1*-dPSTR^Y and p*FIG1*-dPSTR^R (A) p*AGA1*-dPSTR^Y and p*AGA1*-dPSTR^R (B) as function of time. dPSTR^Y is plotted in yellow, right axis, and dPSTR^R in red, left axis.

C. and D. Correlation of the normalized dPSTR nuclear enrichment from all single cells at diferent time points after stimulation in strains carrying two dPSTRs measuring either p*FIG1* (C) or p*AGA1* (D).

E. Intrinsic noise as percent of total noise calculated for p*FIG1* and p*AGA1* at all time points of the time-lapse movie, using the formula from Elowitz *et al.* 2002 (see Methods). The error bars represent the standard deviations calculated from 3 biological replicates.

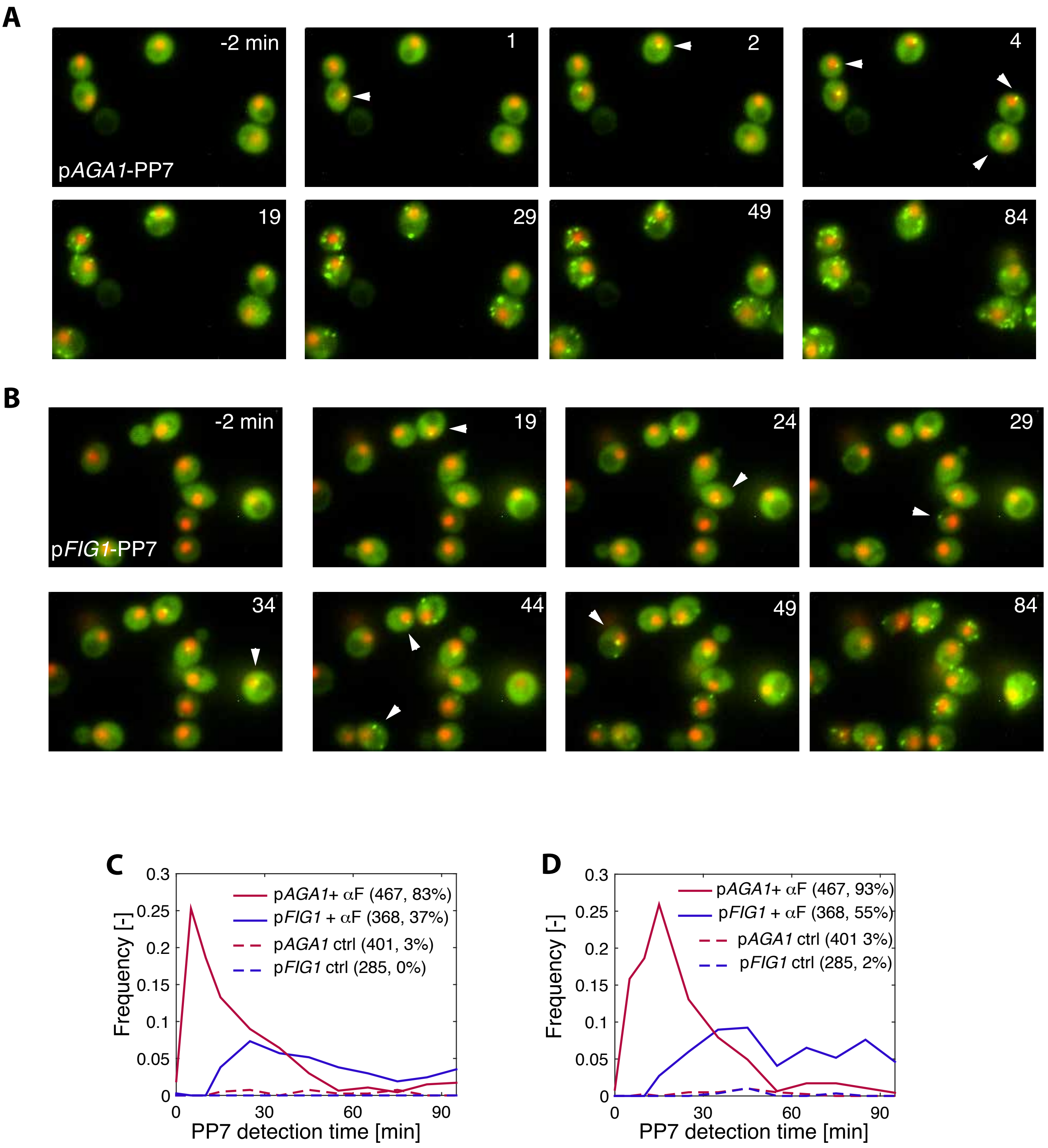

Appendix Figure S7

Timing of mRNA production after pheromone stimulation

A. and B. Images of cells expressing a PP7-2xGFP constitutively and PP7 stem loops under the control of the p*AGA1* (A) or p*FIG1* (B) promoters after pheromone stimulation at time 0. Cells also carry a Hta2-mCherry fusion to visualize the nuclei. Arrows heads indicate when PP7 foci are observed in a cell for the first time.

C. and D. Histograms of the time of PP7 dot detection in the nucleus (C) or in the whole cell (D). The numbers indicate the total number of cells tracked in the time-lapse movie and the percentages represent the proportion of cells where a PP7 focus was detected. The solid lines are cell treated with 1μM α-factor and dashed lines control cells treated with synthetic medium.

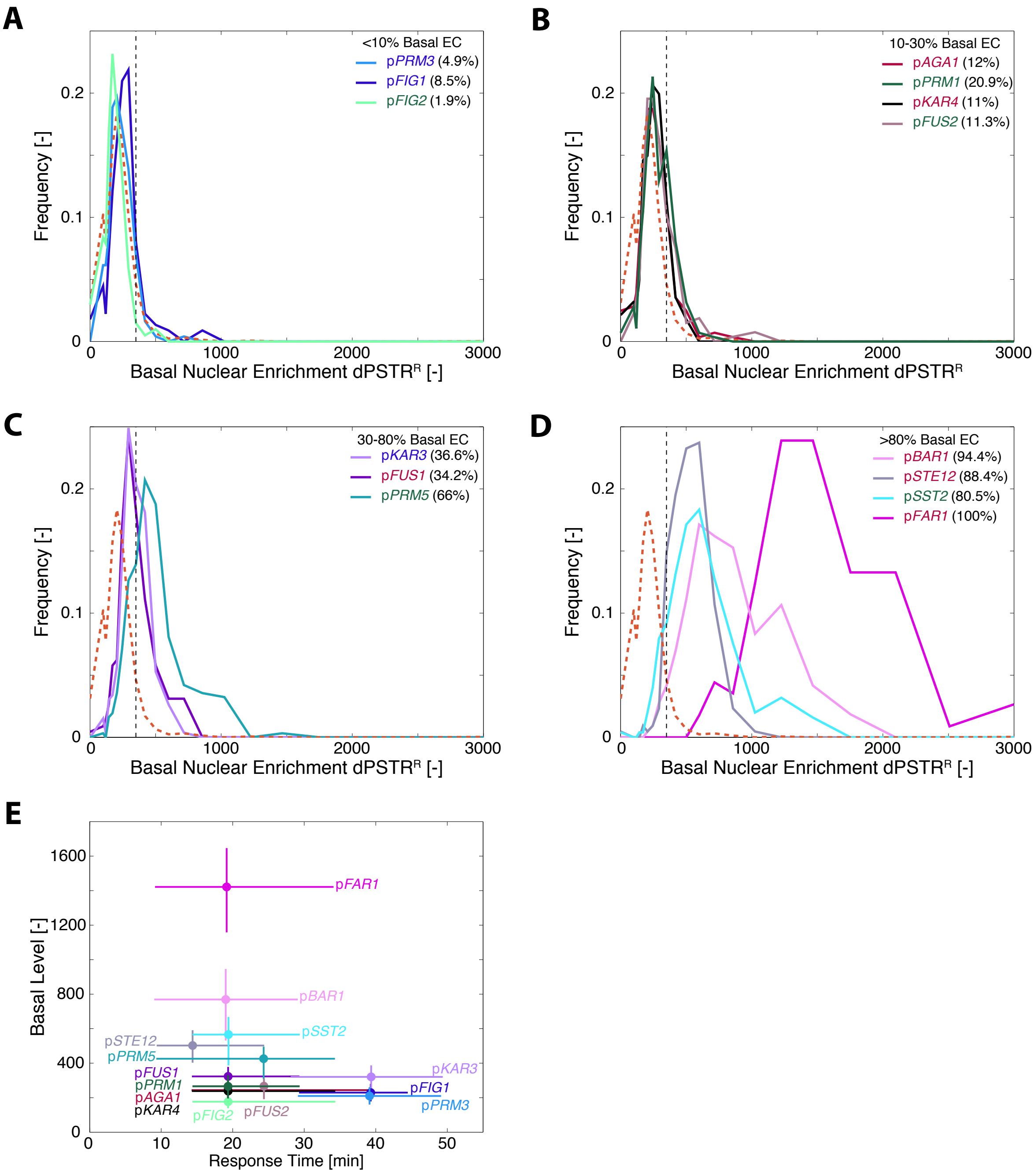

Appendix Figure S8

Basal expression level of mating promoters.

A to D. Histograms of the basal nuclear enrichment of the dPSTR^R are plotted for all cells from the representative experiments chosen for each promoter of the study. The orange dotted line on all plots is the nuclear enrichment observed in a strain carrying only the constitutive part of the dPSTR^R (mCherry-SZ2), without the second half of the construct (MCS2, where the inducible promoter is). From this strain, the threshold for basal enrichment, due to the fluorescent protein itself, was determined (black doted line, N-C=350, N_C=2888), with 5% of false positive cells. Promoters were classified in 4 categories: no basal level (A), low basal level (B), mid basal level (C) and high basal level (D). The percentages in brackets represent the proportion of cells from the entire population of the experiment that express the promoters in vegetative growth (expressing cells, EC). Colors of promoters in the legends indicate the promoter class: early: red; intermediate: green; late: blue.

E. Correlation between the mean basal expression level and the median response time for all promoters in one representative experiment. The lines represent the 25th-75th percentiles. Note that there is no correlation between the basal expression level and the response time.

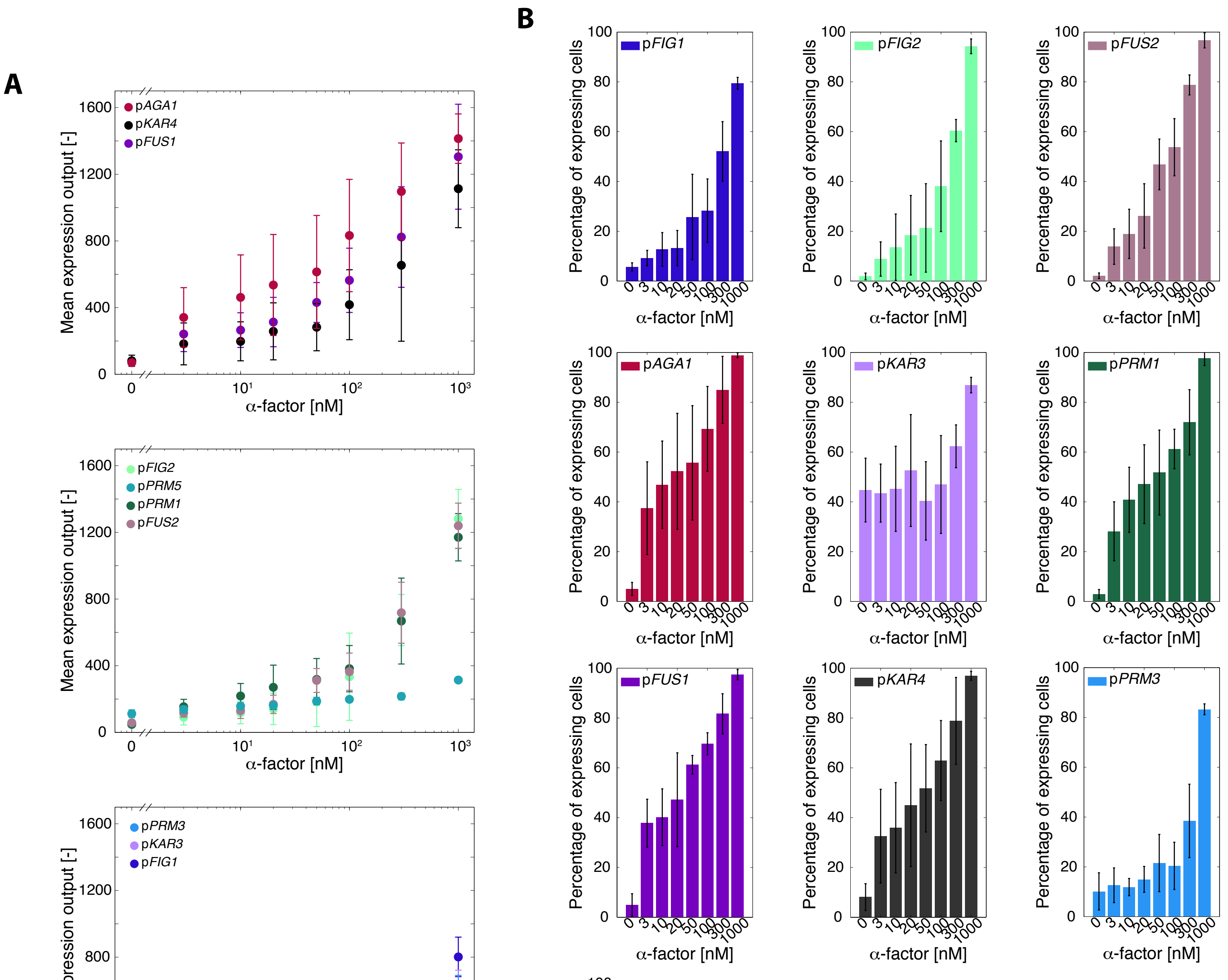

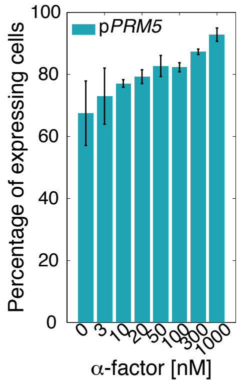

C

$$y = \frac{x^n}{K_d + x^n}$$

Promoter	Kd	n
p*AGA1*	15.79	0.692
p*KAR4*	219.37	1.041
p*FUS1*	67.99	0.867
p*FIG2*	4627.68	1.532
p*PRM5*	24.68	0.676
p*PRM1*	120.55	0.932
p*FUS2*	717.02	1.242
p*PRM3*	38*10^6	2.831
p*KAR3*	38*10^6	2.846
p*FIG1*	38*10^6	2.906

Appendix Figure S9

Dose response various mating-induced promoters.

A. Mean expression output for the indicated promoters in response to different pheromone concentrations. The expression output is defined as the maximal dPSTR nuclear enrichment following stimulation, for all cells of the experiment. Error bars represent the standard deviation of 3 replicates.

B. Percentage of cells expressing the indicated promoter, according to various pheromone concentrations. The error bars represent the standard deviation of three experiments.

C. Table containing the Kd and n coefficients from the fitting of a Hill function on the mean expression outputs data presented in A.

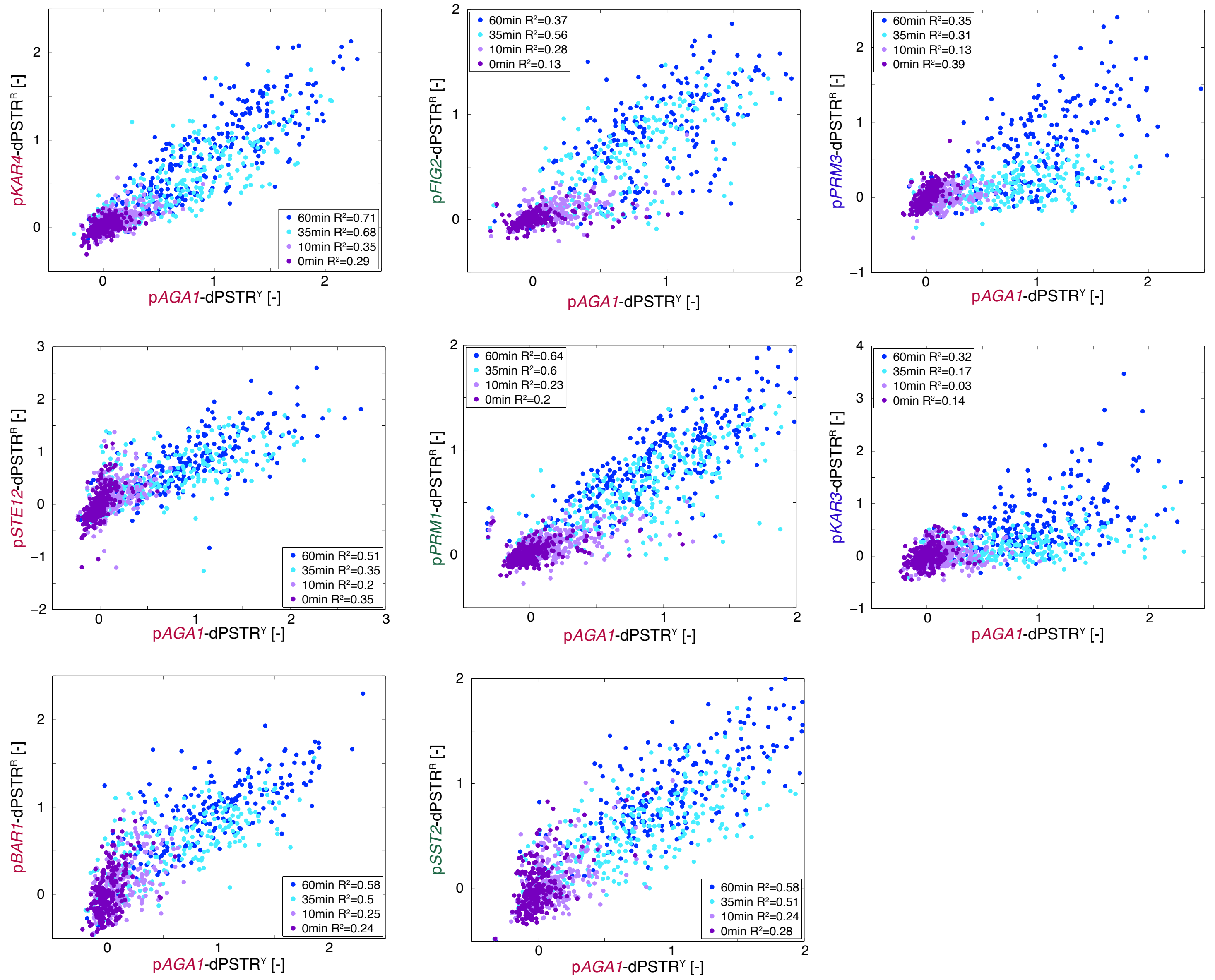

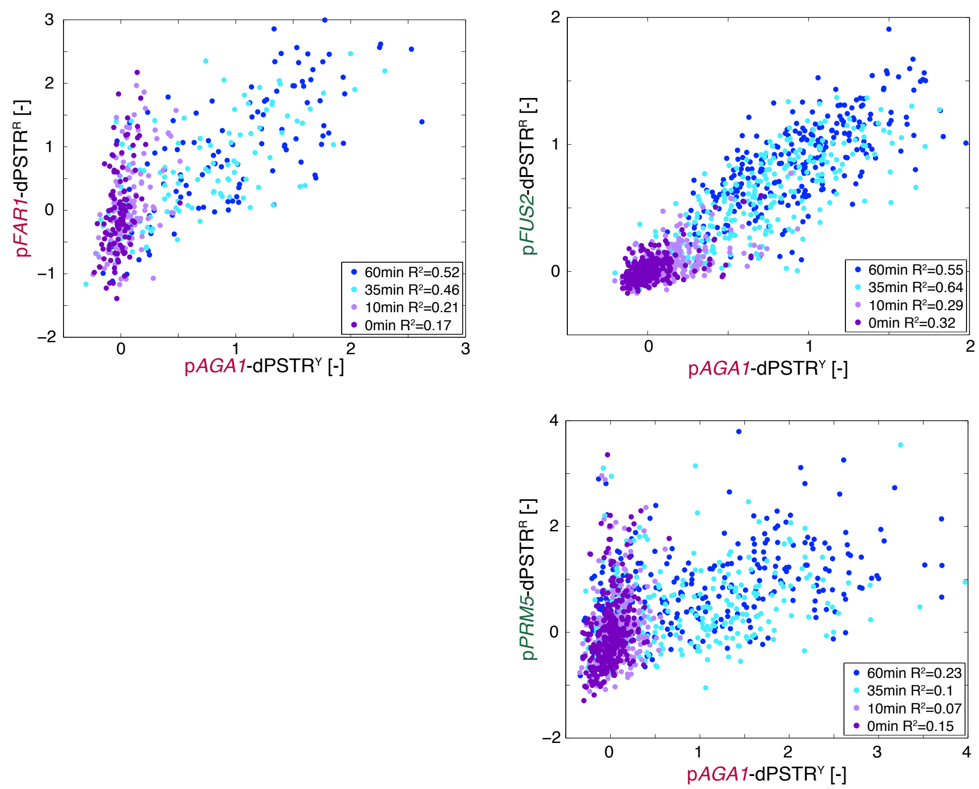

Appendix Figure S10

Dynamic correlation of promoters expression compared to p*AGA1*.

Correlation of normalized dPSTRs nuclear enrichments from all single cells at different time points after stimulation in the strains used in Figure 2A. Promoters in red were categorized as early, in green as intermediate, and in blue as late induced.

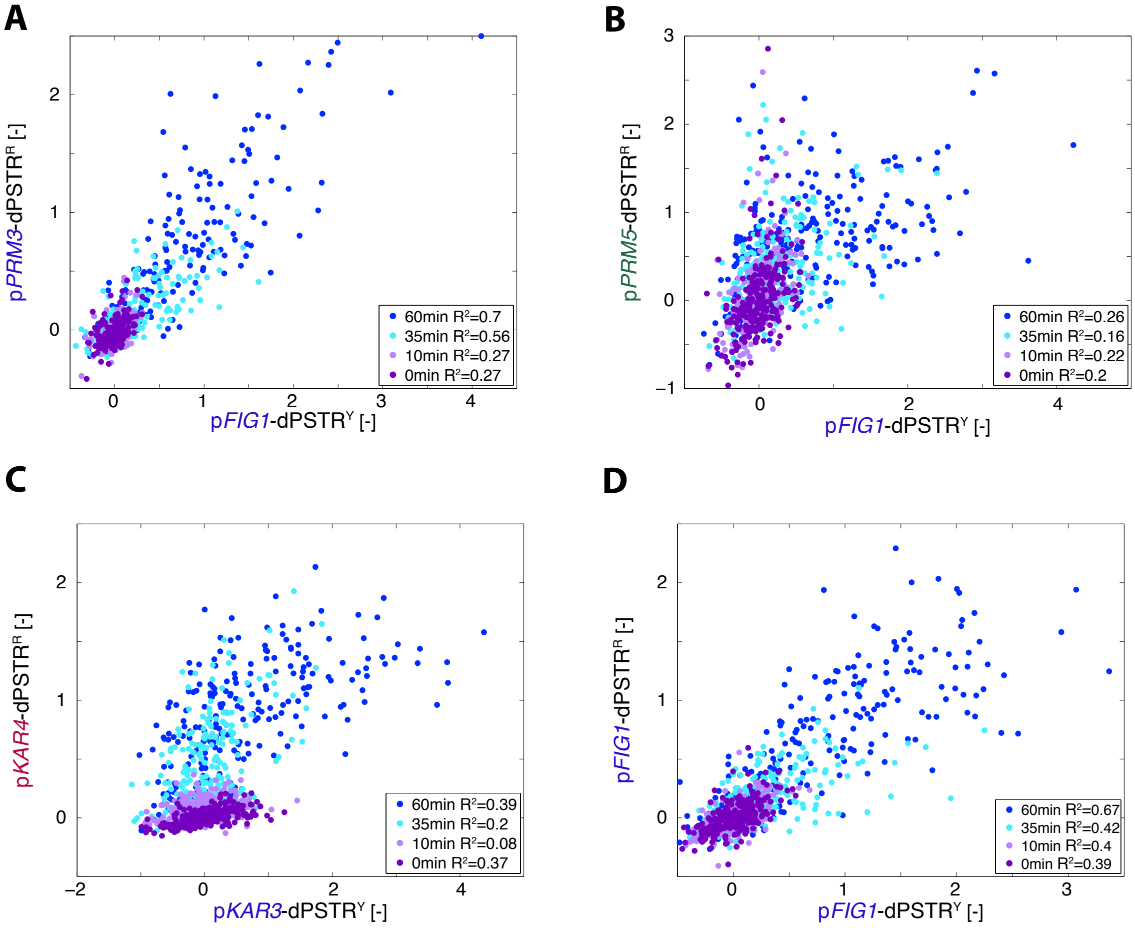

Appendix Figure S11

Dynamic correlations of promoters expression.

A to D. Correlation of normalized dPSTRs nuclear enrichments from all single cells at different time points after stimulation in the strains with various combination of promoters. Promoters in red were categorized as early, in green as intermediate, and in blue as late induced.

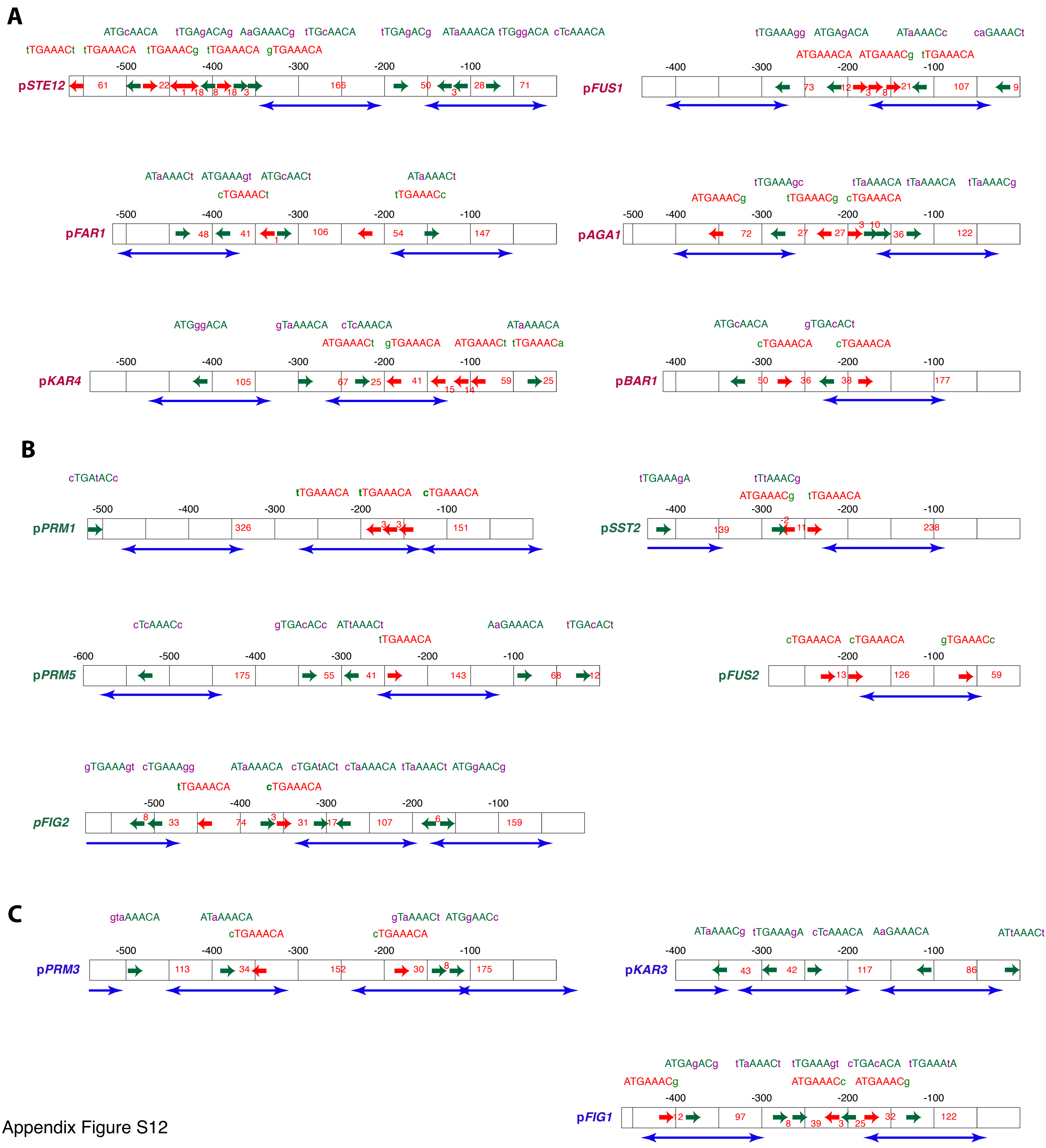

Appendix Figure S12

Maps of promoters analyzed in this study.

All Ste12 binding sites were mapped on the 14 promoters of the study, which are defined as nucleotides from the stop codon of the upstream ORF to the ATG of the gene of interest. Consensus PREs, meaning nTGAAACn, are represented here by red arrows indicating their positions and orientations, with their corresponding sequences above (green lower cases are mutations from the full consensus ATGAAACA). Non consensus PREs as studied by Su *et al.* are representated by green arrows and green sequences. The numbers between the PREs represent the distance in nucleotides separating the last nucleotide of the upstream PRE from the first nucleotide of the downstream PRE. The last number is the relative distance between the last PRE and the start codon. Blue arrows represent nucleosome positioning as extracted from Brogaard *et al* (Nature 2012). Promoters in red were categorized as early, in green as intermediate, and in blue as late induced.

A. Maps of the early promoters.
B. Maps of the intermediate promoters.
C. Maps of the late promoters.

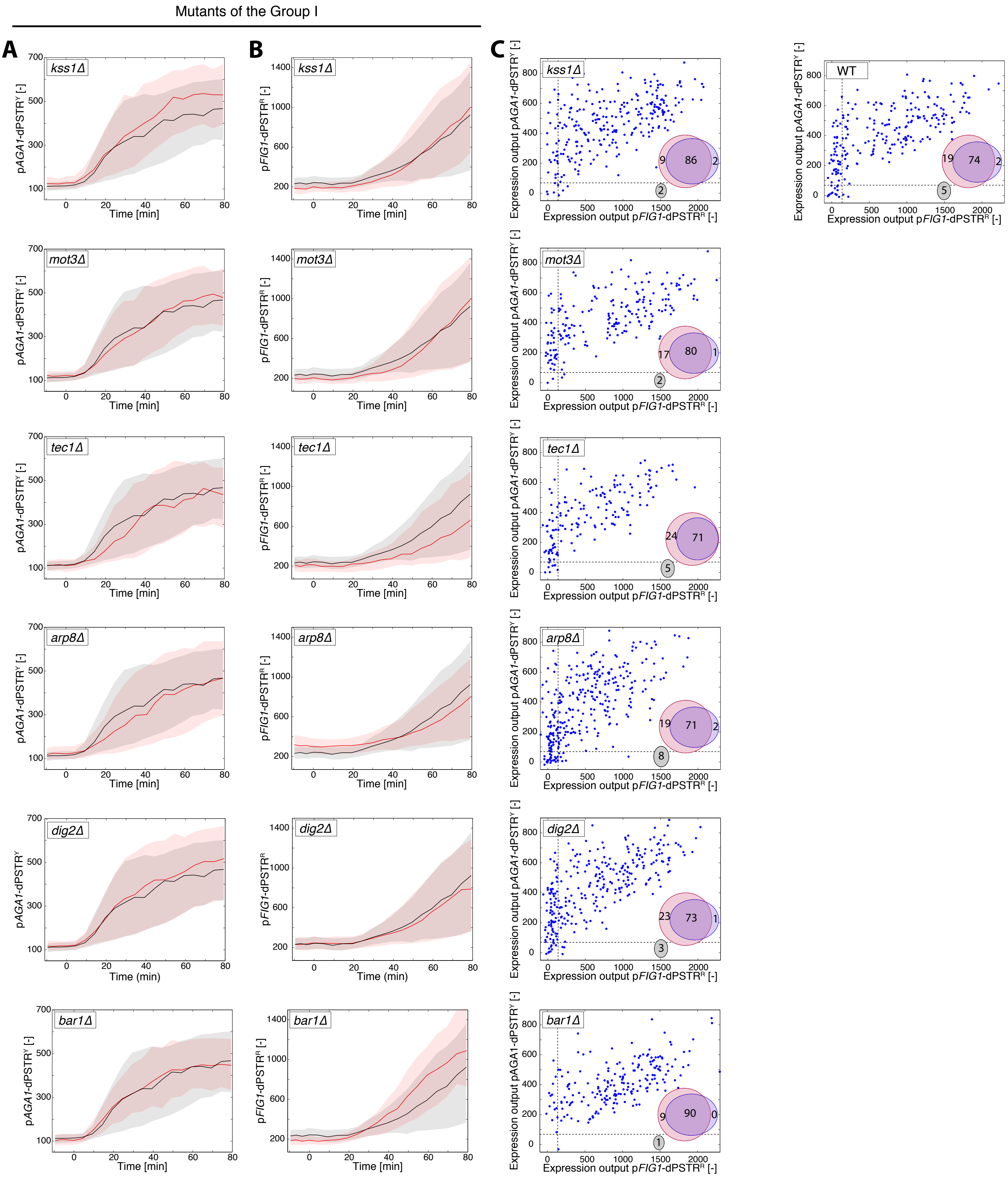

Appendix Figure S13

Mutants of the Group I induce p*FIG1* and p*AGA1* as the WT.

A and B. Nuclear enrichment of the p*AGA1*-dPSTR^{Y} (A) and p*FIG1*-dPSTR^{R} (B) after stimulation by 1μM of pheromone in the indicated mutant. Lines represent the median of either the mutant (red) or the WT strain (black) for one representative experiment, with the shaded area representing the 25th-75th percentile.

C. Correlation of the expression output (maximal dPSTR nuclear enrichment following stimulation) of p*AGA1*-dPSTR^{Y} and p*FIG1*-dPSTR^{R} for all single cells of the experiment, for the indicated strain. Dotted lines represent the threshold of expression (defined as the 20% of the WT mean expression output for each dPSTR). The Venn diagram represents the proportion of cells expressing p*AGA1* (red circle) or p*FIG1* (blue circle) or none of them (black circle).

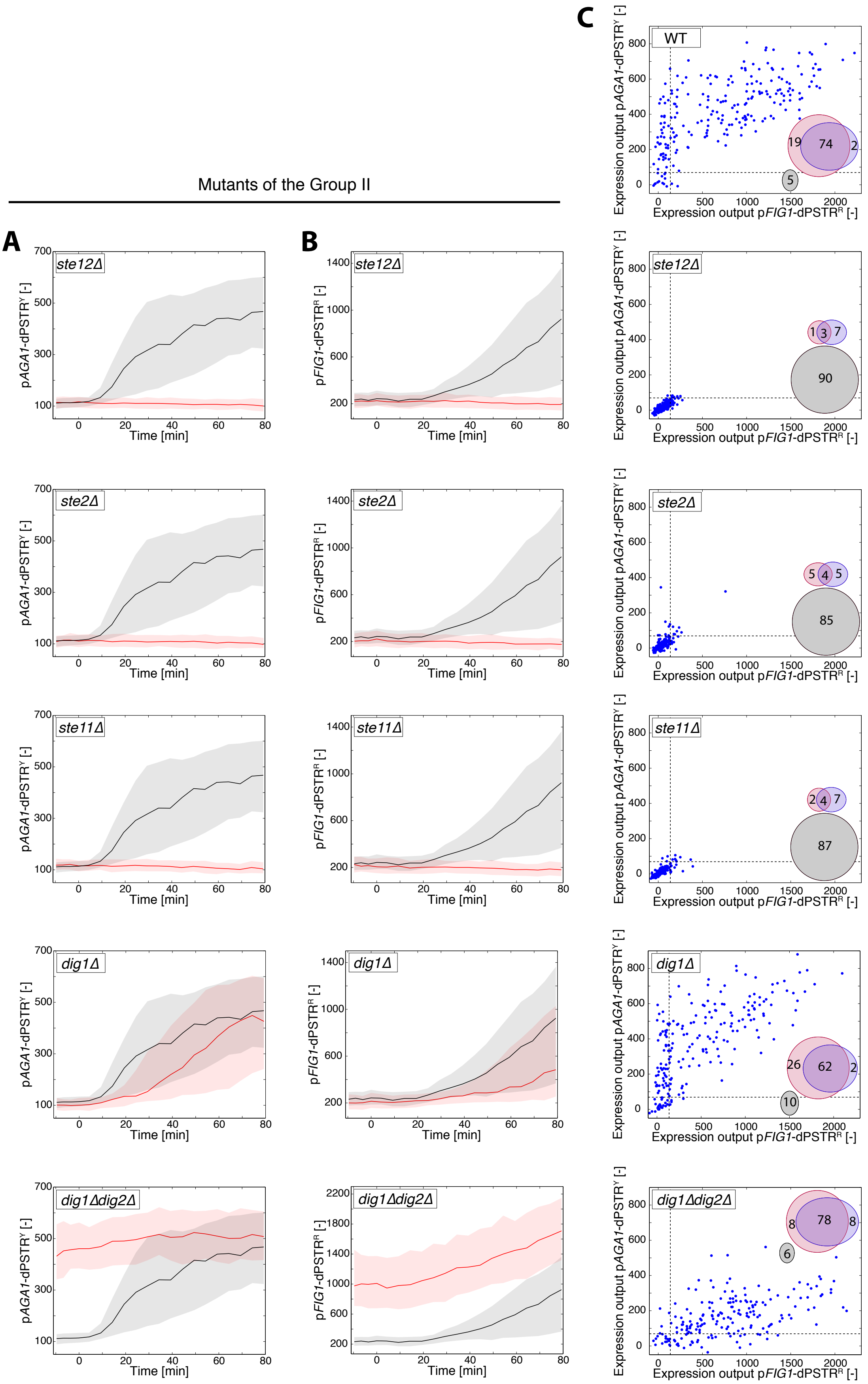

Appendix Figure S14

Mutants of the Group II have an impaired induction of both p*FIG1* and p*AGA1* in a similar manner.

A and B. Nuclear enrichment of the p*AGA1*-dPSTR^{Y} (A) and p*FIG1*-dPSTR^{R} (B) after stimulation by 1μM of pheromone in the indicated mutant. Lines represent the median of either the mutant (red) or the WT strain (black) for one representative experiment, with the shaded area representing the 25th-75th percentile.

C. Correlation of the expression output (maximal dPSTR nuclear enrichment following stimulation) of p*AGA1*-dPSTR^{Y} and p*FIG1*-dPSTR^{R} for all single cells of the experiment, for the indicated strain. Dotted lines represent the threshold of expression (defined as the 20% of the WT mean expression output for each dPSTR). The Venn diagram represents the proportion of cells expressing p*AGA1* (red circle) or p*FIG1* (blue circle) or none of them (black circle).

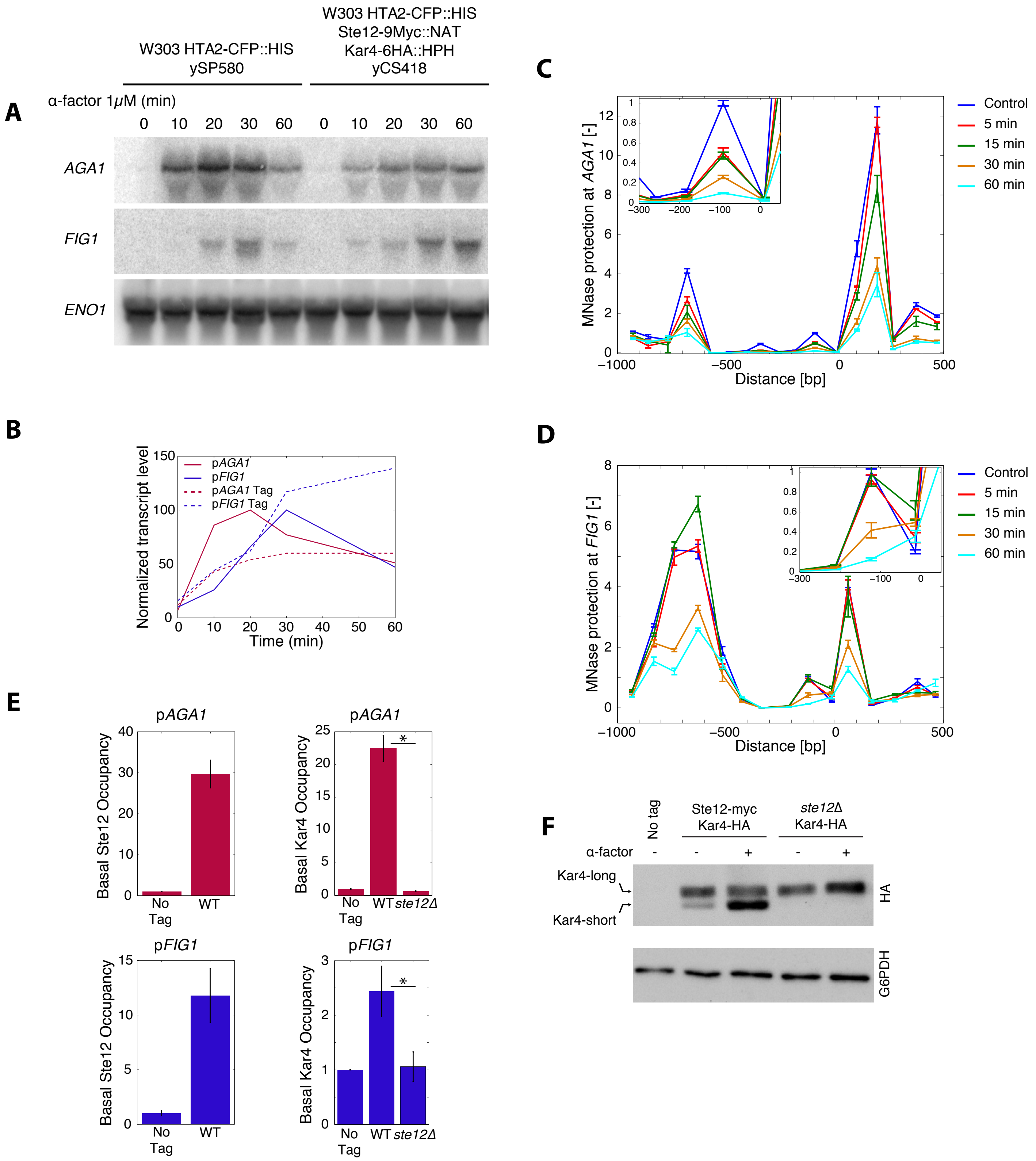

Appendix Figure S15

Biochemistry experiments

A and B. Northern blot detection of mRNAs from *AGA1* and *FIG1* in the reference strain used with the dPSTR (ySP643, left) or the strain carring the tags for biochemistry experiments (yCS418, right). Blot is shown in A, and quantifications normalized on the maximal level of each promoter are shown in B. This is the same image as the one presented in Figure 1H.

C and D. MNase protection assay performed on the endogenous *AGA1* (C) and *FIG1* (D) loci. The intensity of the -1 nucleosome (insert) is plotted in Figure 3C and D.

E. Basal occupancy of Ste12 and Kar4 on the AGA1 and FIG1 promoter relative to a no tag strain. The bars represents the mean of three replicates, and the error bars the standard deviations. Note that Kar4 enrichment at the promoters is dependent on the presence of Ste12 (Student's t-test pVal=6.10^{-7} for p*AGA1* and pVal=$3.9.10^{-3}$ for p*FIG1*).

F. Western blot quantifying Kar4 amount in the strain carrying the two tags or in a *ste12Δ* background.

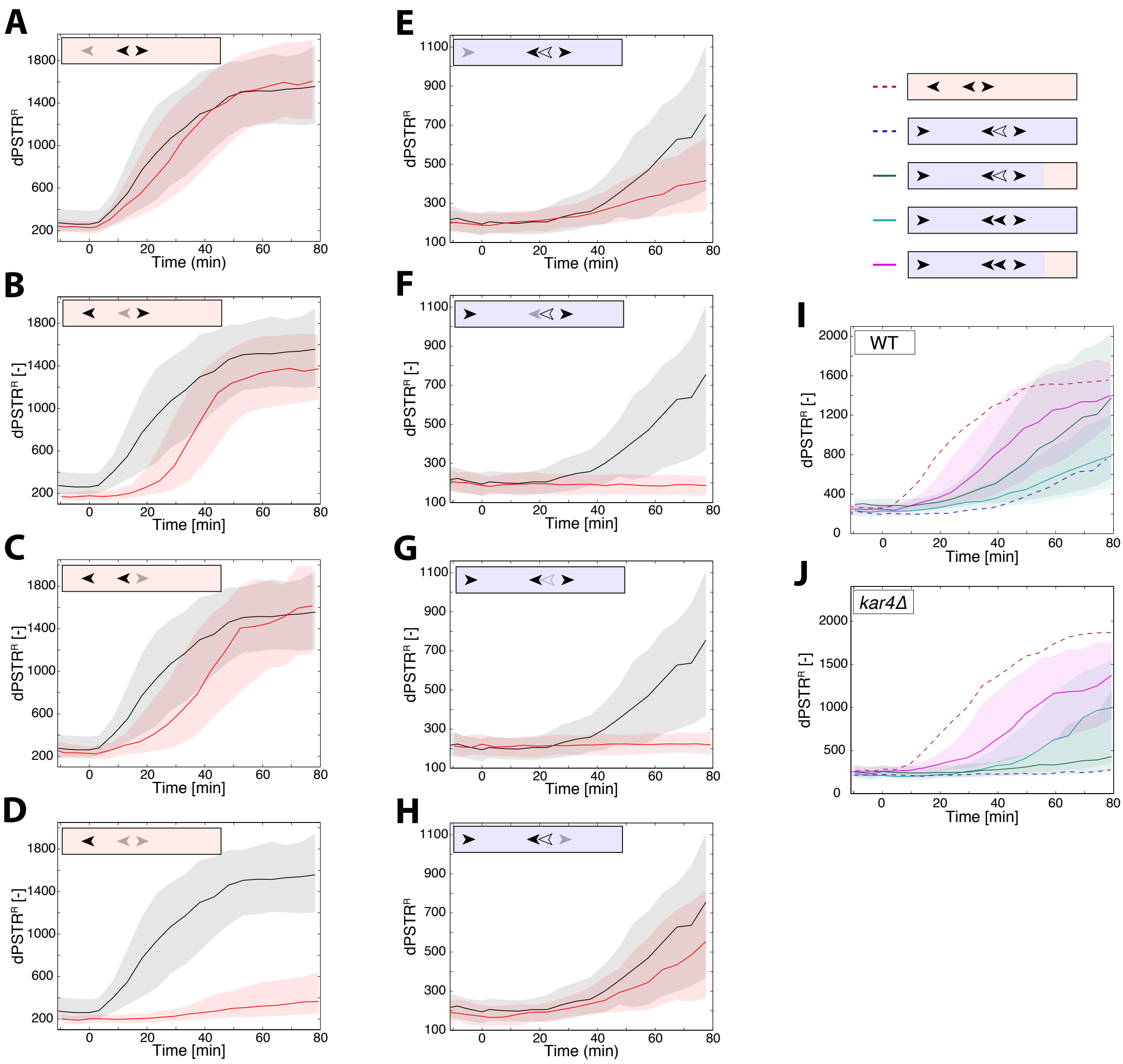

Appendix Figure S16

Mutants of the *AGA1* and *FIG1* promoters help to understand their regulations

A to D. Nuclear enrichment of the p*AGA1*-dPSTR^{R} for mutants (red) compared to the synthetic p*AGA1* (black) with mutation of consensus Ste12 binding sites into restriction sites far from the consensus sequence (See Methods). Mutated sites are indicated in light gray.

E to H. Nuclear enrichment of the p*FIG1*-dPSTR^{R} for mutants (red) compared to the synthetic p*FIG1* (black) with mutation of consensus (E, F, H) or non consensus (G) Ste12 binding sites into restriction sites far from the consensus sequence (See Methods). Mutated sites appear shaded.

F and G. Nuclear enrichment of the dPSTR^{R} for the indicated constructs (top legend) in a WT background (F) or a *kar4Δ* background (G), from the same experiments used to plot Figure 3I. Constructs from top to bottom: p*AGA1*; p*FIG1*; chimera p*FIG1* with the last 150bp from p*AGA1* replacing those from p*FIG1*; p*FIG1* with mutation of a non-consensus binding site into a 4th consensus site, chimera p*FIG1* with 4 consensus binding sites and the last 150bp from p*AGA1*.

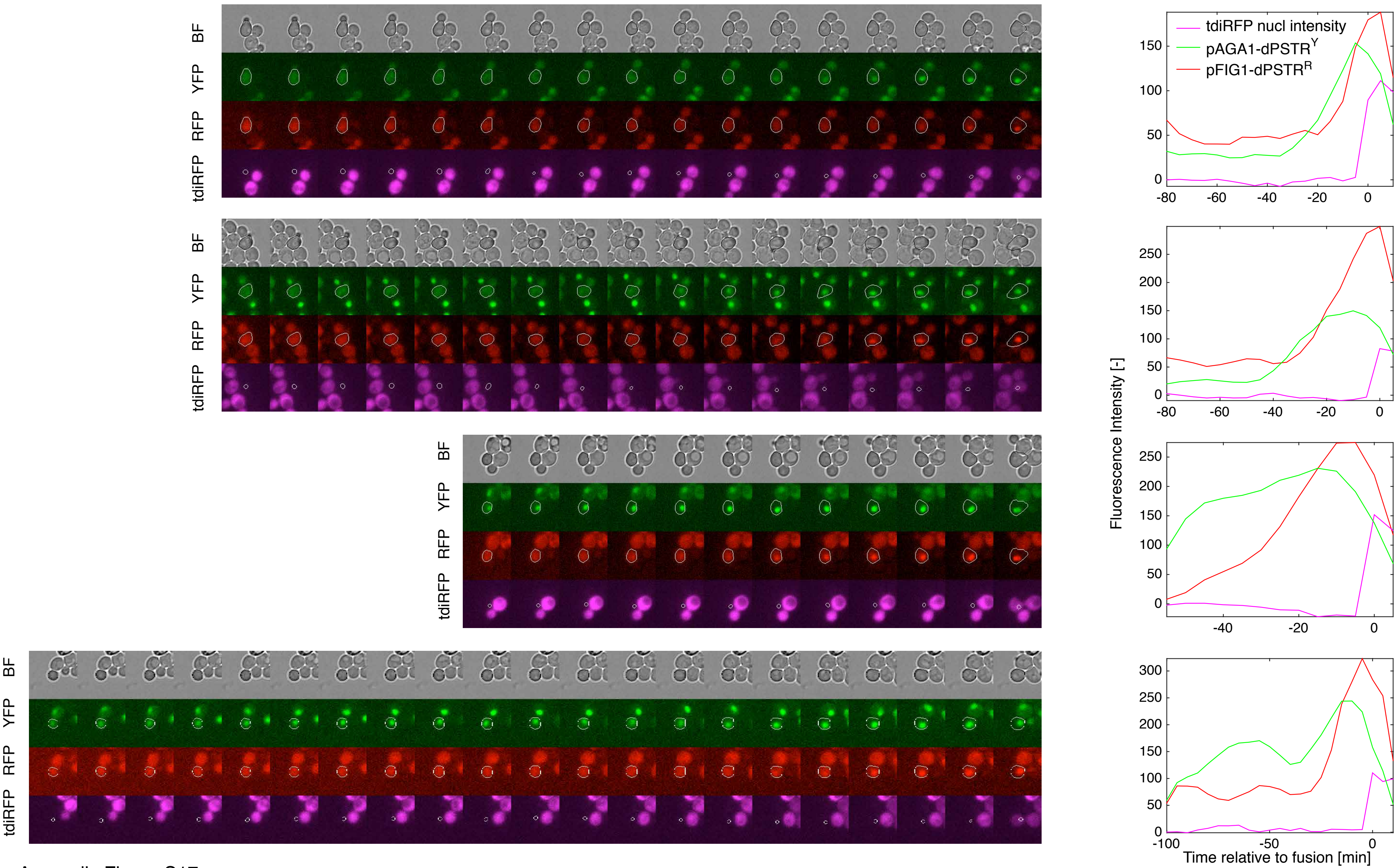

Appendix Figure S17

Detection of fusion events in mating mixtures.

Microscopy images of a mating mixture containing the MAT**a** strain (Hta2-CFP, p*FIG1*-dPSTR^{R} and p*AGA1*-dPSTR^{Y}) and a MATα (cytoplasmic tdiRFP) recorded every 5 minutes. The MAT**a** cells were segmented based on the Hta2-CFP tag and two bright field images to identify the nucleus (white circle in the tdiRFP images) and the cell boundaries (black contour in BF image and white contour in YFP and RFP images). Fusion events are detected by a sudden increase in tdiRFP fluorescence in the nucleus (magenta trace in the left graphs) and define the reference time for the synchronization of the single cell traces. The corresponding dPSTR nuclear enrichments of p*FIG1*-dPSTR^{R} (red) and p*AGA1*-dPSTR^{Y} (green) for these four fusion events are also shown.

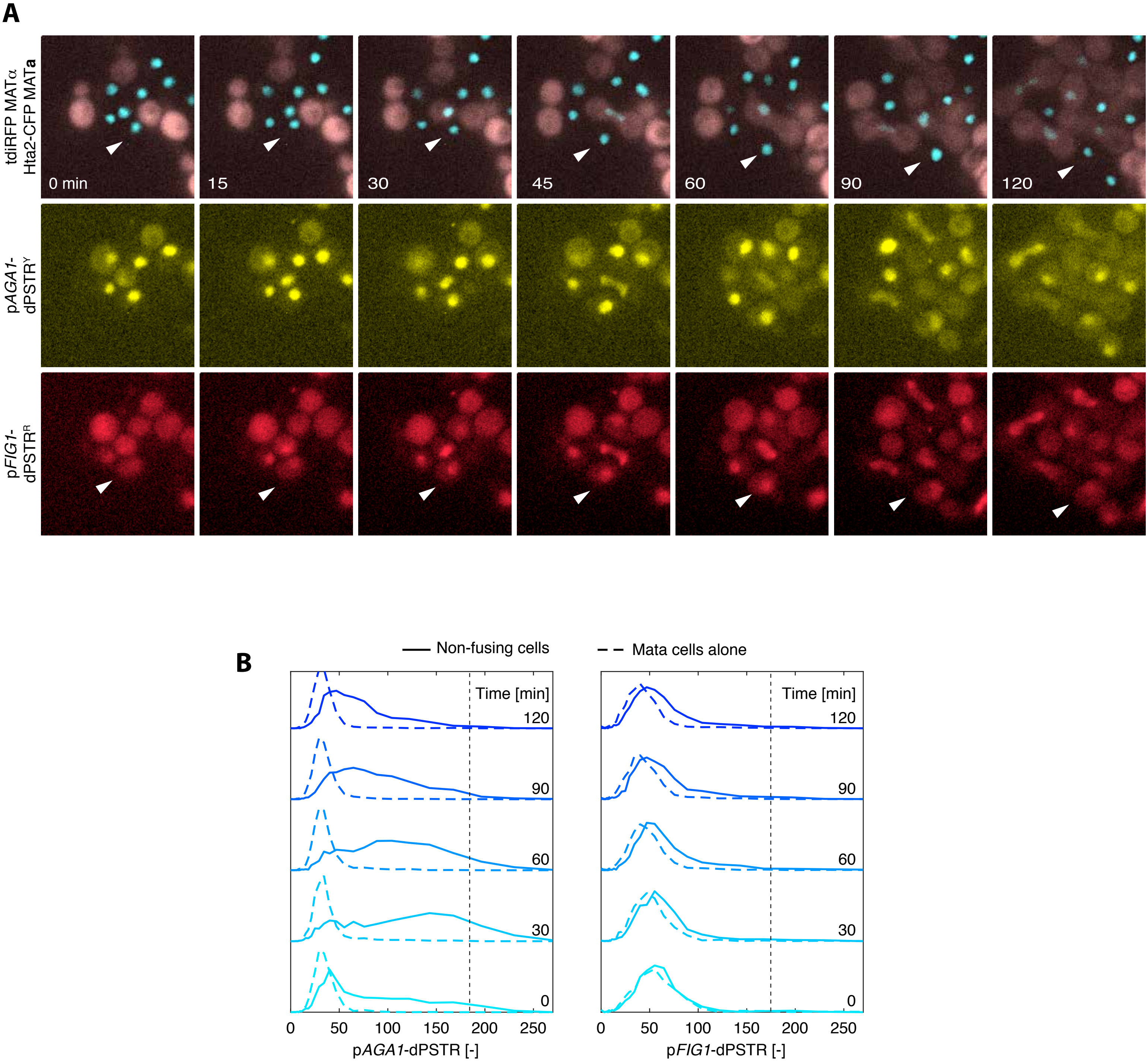

Appendix Figure S18

p*AGA1* and p*FIG1* expression in non-fusing cells.

A. Images representing the rare event of a non-fusing cell (arrow head) that express p*FIG1*. These cells are typically found in the vicinity of a fusing pair and might therefore sense high concentrations of pheromone that trigger p*FIG1* induction.

B. Histograms of p*AGA1* (left panel) and p*FIG1* (right panel) induction at various times after the start of the imaging on pad. The dashed line represent a sample where MAT**a** cells are alone and provide a baseline for basal nuclear enrichment of the reporter in absence of α-factor secretion. The solid line represent histograms of cells that were considered as non-fusing since the tdiRFP fluorescence remained below 1/5 of the threshold used to detect fusion events. In the MAT**a** alone sample this represent 4480 out of 4577 single cell traces and in the mating mixture 2402 cells out of 3046 total cells. The black dashed line represents the maximum of the median of p*AGA1* or p*FIG1* induction in fusing cells.

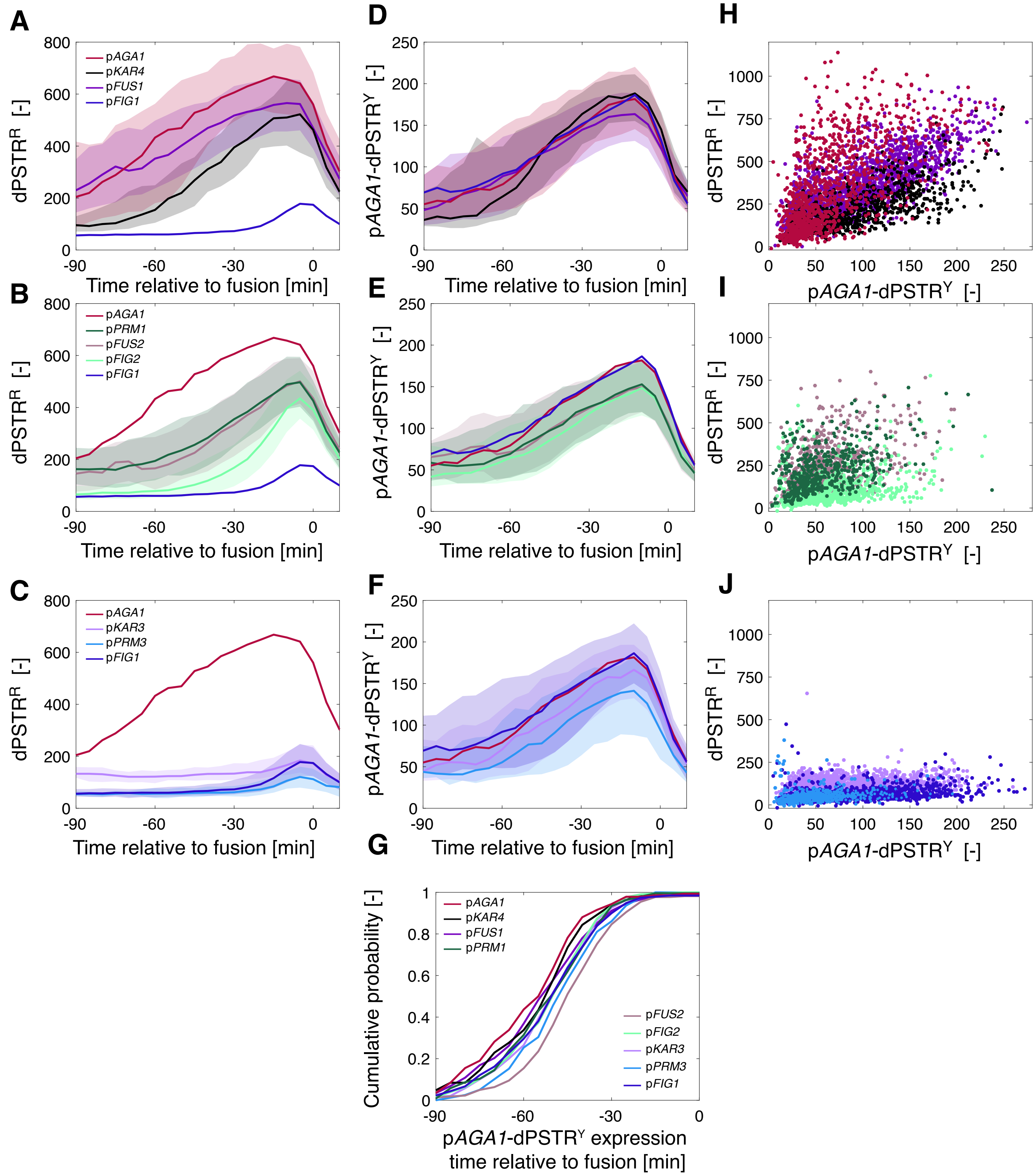

Appendix Figure S19

Dynamics of induction of various promoters relative to fusion time

A to C. Nuclear enrichment of dPSTR^{R} in Mata cells, carrying the p*AGA1*-dPSTR^{Y} mated with a MATα strains. Hundreds of mating pairs were synchronized to their time of fusion (time 0min).

D to F. Nuclear enrichment of p*AGA1*-dPSTR^{Y} quantified in the same cells.

G. Cumulative probability of the response time of the p*AGA1*-dPSTR^{Y} relative to fusion.

H to J. Correlation of the expression output of the dPSTR^{R} relative to the p*AGA1*-dPSTR^{Y}, one hour after the start of the imaging, in cells that do not undergo fusion. Note the lack of expression of the late promoters even in cells inducing strongly the p*AGA1* promoter.

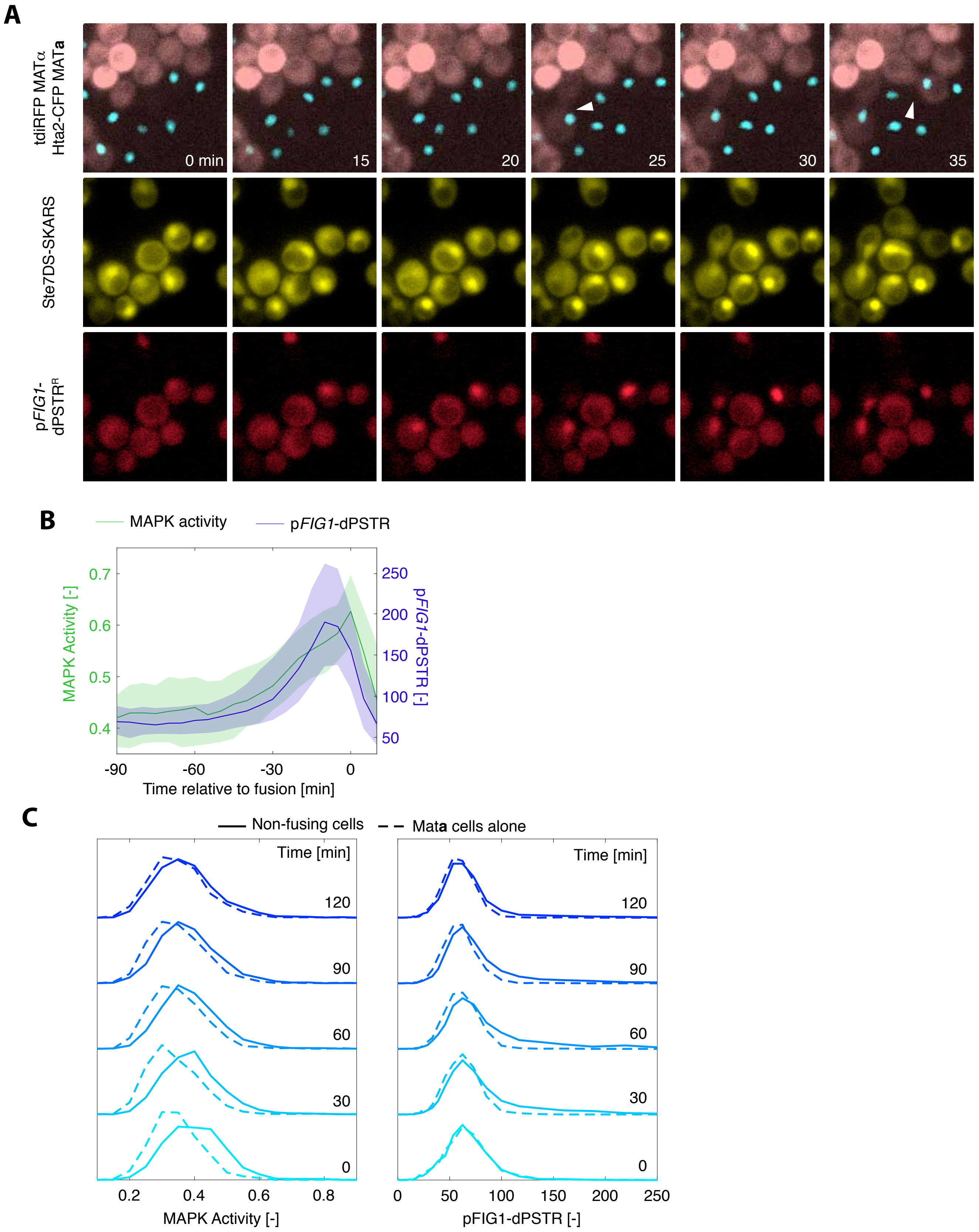

Appendix Figure S20

Evolution of MAPK activity during the mating process.

A. Microscopy images of a mating mixture of a MAT**a** (Hta2-CFP, Ste7$_{DS}$-SKARS^{Y}, p*FIG1*-dPSTR^{R}) and a MATα (cytosolic tdiRFP) taken at various times of the experiment. Fusion events are highlighted by white arrows

B. Median nuclear enrichment of the Ste7$_{DS}$-SKARS^{Y} (green, left axis) and of the p*FIG1*-dPSTR^{R} (blue, right axis) in fusing cells. Single cells traces were synchronized according to their fusion time (time 0) prior to averaging.

C. Histogram of the Ste7$_{DS}$-SKARS^{Y} MAPK activity read-out (left) and the p*FIG1*-dPSTR^{R} nuclear enrichment (right) for cells from the mating mixture were no fusion was detected (solid line) compared to a population of MAT**a** growing on a pad in absence of mating partners (dashed line), at different time points after the start of the experiment.

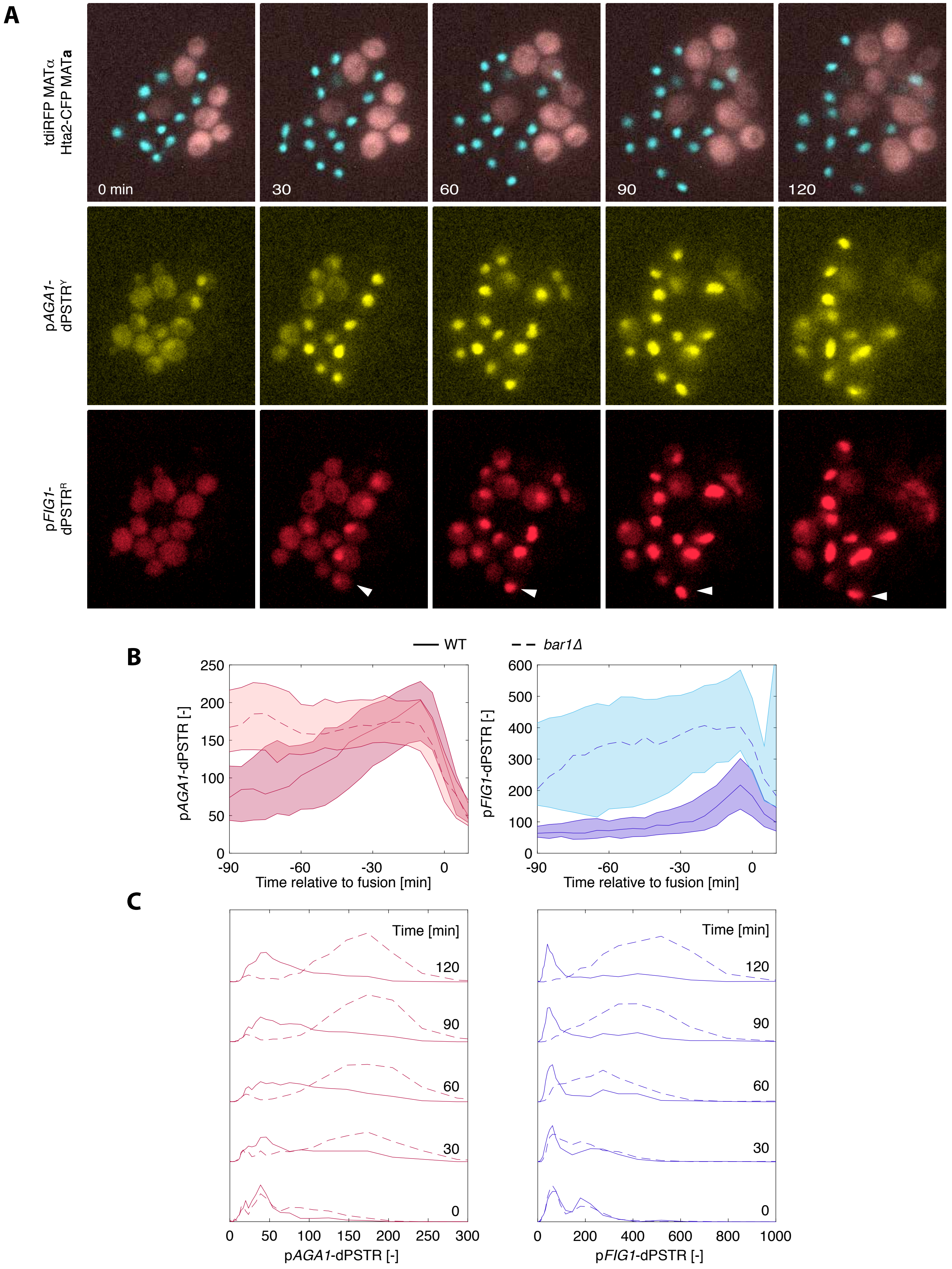

Appendix Figure S21

Gene expression in Mating of *bar1Δ* cells crossed to WT.

A. Images of the fusion process of *bar1Δ* MATa crossed with WT MATα cells. Note the strong induction of p*FIG1* in cells that do not undergo fusion. White arrow highlights one of these non-fusing cell.

B. Nuclear enrichment of the fusing MAT**a** cells in WT (solid line) and *bar1Δ* (dashed line).

C. Histograms of the p*AGA1* and p*FIG1* induction in non-fusing cells for MAT**a** *bar1Δ* cells (dashed line) or MAT**a** WT cells (solid line) in a mating mixture.

Appendix Tables
Appendix Table S1: List of yeast strains used in this study

Strain	Background	Genotype	Plasmid
ySP2	W303	*MATa leu2-3,112 trp1-1 can1-100 ura3-1 ade2-1 his3-11,15*	
ySP122	W303	*MATα leu2-3,112 trp1-1 can1-100 ura3-1 ade2-1 his3-11,15*	
ySP580	ySP2	*HTA2-CFP*	
ySP644	ySP580	*HTA2-CFP*<br>*leu2: pFIG1-dPSTR*$^{Y4-3}$	pSP367
ySP641	ySP580	*HTA2-CFP*<br>*leu2: pAGA1-dPSTR*$^{Y4-3}$	pSP368
ySP671	ySP580	*HTA2-CFP*<br>*ura3: pFIG1-dPSTR*$^{R2-1}$	pSP366
ySP643	ySP641	*HTA2-CFP*<br>*ura3: pFIG1-dPSTR*$^{R2-1}$<br>*leu2: pAGA1-dPSTR*$^{Y4-3}$	pSP366<br>pSP368
yDA146	ySP641	*HTA2-CFP*<br>*ura3: pFAR1-dPSTR*$^{R2-1}$<br>*leu2: pAGA1-dPSTR*$^{Y4-3}$	pDA229<br>pSP368
yDA186	ySP641	*HTA2-CFP*<br>*ura3: pSTE12-dPSTR*$^{R2-1}$<br>*leu2: pAGA1-dPSTR*$^{Y4-3}$	pDA233<br>pSP368
yDA189	ySP641	*HTA2-CFP*<br>*ura3: pFUS1-dPSTR*$^{R2-1}$<br>*leu2: pAGA1-dPSTR*$^{Y4-3}$	pDA239<br>pSP368
yDA198	ySP641	*HTA2-CFP*<br>*ura3: pKAR4-dPSTR*$^{R2-1}$<br>*leu2: pAGA1-dPSTR*$^{Y4-3}$	pDA228<br>pSP368
yDA244	ySP641	*HTA2-CFP*<br>*ura3: pBAR1-dPSTR*$^{R2-1}$<br>*leu2: pAGA1-dPSTR*$^{Y4-3}$	pDA300<br>pSP368
yDA245	ySP641	*HTA2-CFP*<br>*ura3: pSST2-dPSTR*$^{R2-1}$<br>*leu2: pAGA1-dPSTR*$^{Y4-3}$	pDA301<br>pSP368
yDA247	ySP641	*HTA2-CFP*<br>*ura3: pKAR3-dPSTR*$^{R2-1}$<br>*leu2: pAGA1-dPSTR*$^{Y4-3}$	pDA306<br>pSP368
yDA249	ySP641	*HTA2-CFP*<br>*ura3: pPRM3-dPSTR*$^{R2-1}$<br>*leu2: pAGA1-dPSTR*$^{Y4-3}$	pDA305<br>pSP368
ySP664	ySP641	*HTA2-CFP*<br>*ura3: pPRM5-dPSTR*$^{R2-1}$<br>*leu2: pAGA1-dPSTR*$^{Y4-3}$	pSP384<br>pSP368
ySP642	ySP641	*HTA2-CFP*<br>*ura3: pAGA1-dPSTR*$^{R2-1}$<br>*leu2: pAGA1-dPSTR*$^{Y4-3}$	pSP365<br>pSP368
yDA296	ySP641	*HTA2-CFP*<br>*ura3: pFUS2-dPSTR*$^{R2-1}$<br>*leu2: pAGA1-dPSTR*$^{Y4-3}$	pDA334<br>pSP368
yDA302	ySP641	*HTA2-CFP*<br>*ura3: pFIG2-dPSTR*$^{R2-1}$<br>*leu2: pAGA1-dPSTR*$^{Y4-3}$	pDA340<br>pSP368

yDA306	ySP641	*HTA2-CFP*	
		ura3: pPRM1-dPSTR^{R2-1}	pDA338
		leu2: pAGA1-dPSTR^{Y4-3}	pSP368
ySP646	ySP644	*HTA2-CFP*	
		ura3: pFIG1-dPSTR^{R2-1}	pSP366
		leu2: pFIG1-dPSTR^{Y4-3}	pSP367
yDA203	yDA199	*HTA2-CFP*	
		ura3: pKAR4-dPSTR^{R2-1}	pDA228
		leu2: pKAR3-dPSTR^{Y4-3}	pDA268
ySP663	ySP644	*HTA2-CFP*	
		ura3: pPRM5-dPSTR^{R2-1}	pSP384
		leu2: pFIG1-dPSTR^{Y4-3}	pSP367
yDA207	ySP671	*HTA2-CFP*	
		ura3: pFIG1-dPSTR^{R2-1}	pSP366
		leu2: pKAR3-dPSTR^{Y4-3}	pDA268
yDA160	ySP643	*HTA2-CFP*	
		ura3: pFIG1-dPSTR^{R2-1}	pSP366
		leu2: pAGA1-dPSTR^{Y4-3}	pSP368
		kar4::NAT	
yDA163	ySP643	*HTA2-CFP*	
		ura3: pFIG1-dPSTR^{R2-1}	pSP366
		leu2: pAGA1-dPSTR^{Y4-3}	pSP368
		far1::NAT	
yDA218	ySP643	*HTA2-CFP*	
		ura3: pFIG1-dPSTR^{R2-1}	pSP366
		leu2: pAGA1-dPSTR^{Y4-3}	pSP368
		gcn5::NAT	
yDA141	ySP643	*HTA2-CFP*	
		ura3: pFIG1-dPSTR^{R2-1}	pSP366
		leu2: pAGA1-dPSTR^{Y4-3}	pSP368
		kss1::NAT	
yDA161	ySP643	*HTA2-CFP*	
		ura3: pFIG1-dPSTR^{R2-1}	pSP366
		leu2: pAGA1-dPSTR^{Y4-3}	pSP368
		ste11::NAT	
yDA162	ySP643	*HTA2-CFP*	
		ura3: pFIG1-dPSTR^{R2-1}	pSP366
		leu2: pAGA1-dPSTR^{Y4-3}	pSP368
		ste12::NAT	
yDA164	ySP643	*HTA2-CFP*	
		ura3: pFIG1-dPSTR^{R2-1}	pSP366
		leu2: pAGA1-dPSTR^{Y4-3}	pSP368
		ste2::NAT	
yDA142	ySP643	*HTA2-CFP*	
		ura3: pFIG1-dPSTR^{R2-1}	pSP366
		leu2: pAGA1-dPSTR^{Y4-3}	pSP368
		fus3::NAT	
yDA208	ySP643	*HTA2-CFP*	
		ura3: pFIG1-dPSTR^{R2-1}	pSP366
		leu2: pAGA1-dPSTR^{Y4-3}	pSP368
		mot3::NAT	
yDA151	ySP643	*HTA2-CFP*	
		ura3: pFIG1-dPSTR^{R2-1}	pSP366
		leu2: pAGA1-dPSTR^{Y4-3}	pSP368
		dig1::NAT	

yDA159	ySP643	*HTA2-CFP*<br>*ura3: pFIG1-dPSTR*$^{R2-1}$<br>*leu2: pAGA1-dPSTR*$^{Y4-3}$<br>*tec1::NAT*	 pSP366<br>pSP368
yDA219	ySP643	*HTA2-CFP*<br>*ura3: pFIG1-dPSTR*$^{R2-1}$<br>*leu2: pAGA1-dPSTR*$^{Y4-3}$<br>*arp8::NAT*	 pSP366<br>pSP368
ySP692	ySP643	*HTA2-CFP*<br>*ura3: pFIG1-dPSTR*$^{R2-1}$<br>*leu2: pAGA1-dPSTR*$^{Y4-3}$<br>*bar1::NAT*	 pSP366<br>pSP368
yDA305	ySP643	*HTA2-CFP*<br>*ura3: pFIG1-dPSTR*$^{R2-1}$<br>*leu2: pAGA1-dPSTR*$^{Y4-3}$<br>*dig2::NAT*<br>*dig1::KAN*	 pSP366<br>pSP368
yDA317	ySP643	*HTA2-CFP*<br>*ura3: pFIG1-dPSTR*$^{R2-1}$<br>*leu2: pAGA1-dPSTR*$^{Y4-3}$<br>*dig2::NAT*	 pSP366<br>pSP368
yDA316	yDA249	*HTA2-CFP*<br>*ura3: pPRM3-dPSTR*$^{R2-1}$<br>*leu2: pAGA1-dPSTR*$^{Y4-3}$<br>*kar4::NAT*	 pDA305<br>pSP368
yDA348	ySP642	*HTA2-CFP*<br>*ura3: pAGA1-dPSTR*$^{R2-1}$<br>*leu2: pAGA1-dPSTR*$^{Y4-3}$<br>*kar4::NAT*	 pSP365<br>pSP368
yDA319	yDA296	*HTA2-CFP*<br>*ura3: pFUS2-dPSTR*$^{R2-1}$<br>*leu2: pAGA1-dPSTR*$^{Y4-3}$<br>*kar4::NAT*	 pDA334<br>pSP368
yDA320	yDA302	*HTA2-CFP*<br>*ura3: pFIG2-dPSTR*$^{R2-1}$<br>*leu2: pAGA1-dPSTR*$^{Y4-3}$<br>*kar4::NAT*	 pDA340<br>pSP368
yDA322	yDA306	*HTA2-CFP*<br>*ura3: pPRM1-dPSTR*$^{R2-1}$<br>*leu2: pAGA1-dPSTR*$^{Y4-3}$<br>*kar4::NAT*	 pDA338<br>pSP368
ySP614	ySP580	*HTA2-CFP*<br>*ura3: pFIG1-dPSTR*$^{R3-4}$<br>*trp3: Ste7*$_{DS}$*-SKARS*^{Y}	 pSP343<br>pED73
ySP615	ySP580	*HTA2-CFP*<br>*ura3: pAGA1-dPSTR*$^{R3-4}$<br>*trp3: Ste7*$_{DS}$*-SKARS*^{Y}	 pSP350<br>pED73
yCS418	ySP580	*HTA2-CFP*<br>*Ste12-9myc::NAT*<br>*Kar4-6HA::HPH*	
yDA228	ySP641	*HTA2-CFP*<br>*ura3: pFIG1*AGA1 -dPSTR*$^{R2-1}$<br>*leu2: pAGA1-dPSTR*$^{Y4-3}$	 pDA284<br>pSP368
yDA313	ySP644	*HTA2-CFP*<br>*ura3: pFIG1syn_PREII*^{nc}*_to_consensus-*	 pDA344

		dPSTR^{R2-1}<br>leu2: pFIG1-dPSTR^{Y4-3}	 pSP367
yDA352	ySP644	HTA2-CFP<br>ura3:<br>pFIG1syn_PREII^{nc}_to_consensus*AGA1 -<br>dPSTR^{R2-1}<br>leu2: pFIG1-dPSTR^{Y4-3}	 pDA387<br>pSP367
yDA234	yDA228	HTA2-CFP<br>ura3: pFIG1*AGA1 -dPSTR^{R2-1}<br>leu2: pAGA1-dPSTR^{Y4-3}<br>kar4::NAT	 pDA284<br>pSP368
yDA326	yDA313	HTA2-CFP<br>ura3: pFIG1syn_PREII^{nc}_to_consensus-<br>dPSTR^{R2-1}<br>leu2: pFIG1-dPSTR^{Y4-3}<br>kar4::NAT	 pDA344<br><br>pSP367
yDA256	ySP644	HTA2-CFP<br>ura3: pFIG1syn_ΔPREI-dPSTR^{R2-1}<br>leu2: pFIG1-dPSTR^{Y4-3}	 pDA286<br>pSP367
yDA258	ySP644	HTA2-CFP<br>ura3: pFIG1syn_ΔPREIII-dPSTR^{R2-1}<br>leu2: pFIG1-dPSTR^{Y4-3}	 pDA288<br>pSP367
yDA238	ySP641	HTA2-CFP<br>ura3: pAGA1syn_ΔPREI-dPSTR^{R2-1}<br>leu2: pAGA1-dPSTR^{Y4-3}	 pDA289<br>pSP368
yDA314	ySP644	HTA2-CFP<br>ura3: pFIG1syn_ΔPREII^{nc} -dPSTR^{R2-1}<br>leu2: pFIG1-dPSTR^{Y4-3}	 pDA345<br>pSP367
yDA358	yDA352	HTA2-CFP<br>ura3:<br>pFIG1syn_PREII^{nc}_to_consensus*AGA1 -<br>dPSTR^{R2-1}<br>leu2: pFIG1-dPSTR^{Y4-3}<br>kar4::NAT	 pDA387<br>pSP367
ySP711	ySP122	ura3: pGDP-tdiRFP	pSP397
ySP377	W303	HTA2-mCherry:ura3<br>trp:pMET25-PP7-2xGFP<br>glt1: HIS3-pFIG1-24xPP7-SL	 pSP268<br>pSP267
ySP725	W303	HTA2-mCherry:ura3<br>trp:pMET25-PP7-2xGFP<br>glt1: HIS3-pAGA1-24xPP7-SL	 pSP268<br>pSP409
ySP645	ySP580	HTA2-CFP<br>ura3: pAGA1-dPSTR^{R2-1}<br>leu2: pFIG1-dPSTR^{Y4-3}	 pSP365<br>pSP367

* The numbers in the superscript dPSTR^{R2-1} indicate the pair of SynZip used, and the letter the color of the fluorescent protein (R for mCherry and Y for MCitrine).

Appendix Table S2: List of plasmids used in this study

Plasmid	MCS1	MCS2	Backbone	Strain
pSP368	p*RPL24B*-MCitrine-SynZip4-t*NUP53*	p*AGA1*-2xSv40NLS-SynZip3-t*CYC1*	pSIVL	ySP641
pSP367	p*RPL24B*-MCitrine-SynZip4-t*NUP53*	p*FIG1*-2xSv40NLS-SynZip3-t*CYC1*	pSIVL	ySP644
pDA268	p*RPL24B*-MCitrine-SynZip4-t*NUP53*	p*KAR3*-2xSv40NLS-SynZip3-t*CYC1*	pSIVL	ySP641
pSP366	p*RPL24A*-mCherry-SynZip2-tSIF2	p*FIG1*-2xSv40NLS-SynZip1-t*CYC1*	pSIVU	ySP643
pSP365	p*RPL24A*-mCherry-SynZip2-tSIF2	p*AGA1*-2xSv40NLS-SynZip1-t*CYC1*	pSIVU	ySP642
pDA229	p*RPL24A*-mCherry-SynZip2-tSIF2	p*FAR1*-2xSv40NLS-SynZip1-t*CYC1*	pSIVU	yDA146
pDA233	p*RPL24A*-mCherry-SynZip2-tSIF2	p*STE12*-2xSv40NLS-SynZip1-t*CYC1*	pSIVU	yDA186
pDA239	p*RPL24A*-mCherry-SynZip2-tSIF2	p*FUS1*-2xSv40NLS-SynZip1-t*CYC1*	pSIVU	yDA189
pDA228	p*RPL24A*-mCherry-SynZip2-tSIF2	p*KAR4*-2xSv40NLS-SynZip1-t*CYC1*	pSIVU	yDA198
pDA300	p*RPL24A*-mCherry-SynZip2-tSIF2	p*BAR1*-2xSv40NLS-SynZip1-t*CYC1*	pSIVU	yDA244
pDA301	p*RPL24A*-mCherry-SynZip2-tSIF2	p*SST2*-2xSv40NLS-SynZip1-t*CYC1*	pSIVU	yDA245
pDA306	p*RPL24A*-mCherry-SynZip2-tSIF2	p*KAR3*-2xSv40NLS-SynZip1-t*CYC1*	pSIVU	yDA247
pDA305	p*RPL24A*-mCherry-SynZip2-tSIF2	p*PRM3*-2xSv40NLS-SynZip1-t*CYC1*	pSIVU	yDA249
pSP384	p*RPL24A*-mCherry-SynZip2-tSIF2	p*PRM5*-2xSv40NLS-SynZip1-t*CYC1*	pSIVU	ySP664
pDA334	p*RPL24A*-mCherry-SynZip2-tSIF2	p*FUS2*-2xSv40NLS-SynZip1-t*CYC1*	pSIVU	yDA296
pDA340	p*RPL24A*-mCherry-SynZip2-tSIF2	p*FIG2*-2xSv40NLS-SynZip1-t*CYC1*	pSIVU	yDA302
pDA338	p*RPL24A*-mCherry-SynZip2-tSIF2	p*PRM1*-2xSv40NLS-SynZip1-t*CYC1*	pSIVU	yDA306
pDA284	p*RPL24A*-mCherry-SynZip2-tSIF2	*pFIG1*AGA1 -* 2xSv40NLS-SynZip1-t*CYC1*	pSIVU	yDA228
pDA344	p*RPL24A*-mCherry-SynZip2-tSIF2	*pFIG1syn_PREII*^{nc}*_to_consensus*-2xSv40NLS-SynZip1-t*CYC1*	pSIVU	yDA313
pDA387	p*RPL24A*-mCherry-SynZip2-tSIF2	*pFIG1 PREII*^{nc}*_to_consensus* **AGA1* -2xSv40NLS-SynZip1-t*CYC1*	pSIVU	yDA352
pSP397	p*GPD*-tdiRFP		pRS306	ySP711
pSP268	p*MET* PP7-2xGFP		pRS304	ySP377, ySP725
pSP267	p*FIG1* 24xPP7SL		pHIS	ySP377
pSP409	p*AGA1* 24xPP7SL		pHIS	ySP725

Appendix Table S3: Number of single cell used to generate each figure

Fig. Name	Strain	Reporters	Number of cells in the experiment	Number of cells in the plot
**Fig. 1C**	ySP614	*pFIG1-dPSTR*$^{R3-4}$<br>*Ste7*$_{DS}$*-SKARS*^{Y}	236	236
**Fig. 1D**	ySP615	*pAGA1-dPSTR*$^{R3-4}$<br>*Ste7*$_{DS}$*-SKARS*^{Y}	291	291
**Fig. 1F**	ySP643	*pFIG1-dPSTR*$^{R2-1}$<br>*pAGA1-dPSTR*$^{Y4-3}$	224	p*AGA1*: 208<br>p*FIG1*: 169
**Fig. 1F (inset)**	ySP643	*pFIG1-dPSTR*$^{R2-1}$<br>*pAGA1-dPSTR*$^{Y4-3}$	224	167
**Fig. 1G**	ySP643	*pFIG1-dPSTR*$^{R2-1}$<br>*pAGA1-dPSTR*$^{Y4-3}$	224	224
**Fig. 2A**	ySP642	*pAGA1-dPSTR*$^{R2-1}$<br>*pAGA1-dPSTR*$^{Y4-3}$	283	281
**Fig. 2A**	yDA189	*pFUS1-dPSTR*$^{R2-1}$<br>*pAGA1-dPSTR*$^{Y4-3}$	225	220
**Fig. 2A**	yDA244	*pBAR1-dPSTR*$^{R2-1}$<br>*pAGA1-dPSTR*$^{Y4-3}$	216	213
**Fig. 2A**	yDA296	*pFUS2-dPSTR*$^{R2-1}$<br>*pAGA1-dPSTR*$^{Y4-3}$	267	263
**Fig. 2A**	yDA306	*pPRM1-dPSTR*$^{R2-1}$<br>*pAGA1-dPSTR*$^{Y4-3}$	289	284
**Fig. 2A**	yDA302	*pFIG2-dPSTR*$^{R2-1}$<br>*pAGA1-dPSTR*$^{Y4-3}$	207	195
**Fig. 2A**	yDA245	*pSST2-dPSTR*$^{R2-1}$<br>*pAGA1-dPSTR*$^{Y4-3}$	251	248
**Fig. 2A**	yDA198	*pKAR4-dPSTR*$^{R2-1}$<br>*pAGA1-dPSTR*$^{Y4-3}$	281	267
**Fig. 2A**	yDA146	*pFAR1-dPSTR*$^{R2-1}$<br>*pAGA1-dPSTR*$^{Y4-3}$	113	96
**Fig. 2A**	ySP643	*pFIG1-dPSTR*$^{R2-1}$<br>*pAGA1-dPSTR*$^{Y4-3}$	224	168
**Fig. 2A**	yDA249	*pPRM3-dPSTR*$^{R2-1}$<br>*pAGA1-dPSTR*$^{Y4-3}$	244	191
**Fig. 2A**	yDA247	*pKAR3-dPSTR*$^{R2-}$<br>*pAGA1-dPSTR*$^{Y4-3}$	265	235
**Fig. 2A**	yDA186	*pSTE12-dPSTR*$^{R2-1}$<br>*pAGA1-dPSTR*$^{Y4-3}$	215	205
**Fig. 2A**	ySP663	*pPRM5-dPSTR*$^{R2-1}$<br>*pAGA1-dPSTR*$^{Y4-3}$	309	279
**Fig. 2B**	yDA189	*pFUS1-dPSTR*$^{R2-1}$<br>*pAGA1-dPSTR*$^{Y4-3}$	255	213
**Fig. 2B**	yDA296	*pFUS2-dPSTR*$^{R2-1}$<br>*pAGA1-dPSTR*$^{Y4-3}$	267	263
**Fig. 2B**	ySP643	*pFIG1-dPSTR*$^{R2-1}$<br>*pAGA1-dPSTR*$^{Y4-3}$	224	165
**Fig. 2C**	ySP642	*pAGA1-dPSTR*$^{R2-1}$<br>*pAGA1-dPSTR*$^{Y4-3}$	283	283
**Fig. 2C**	yDA189	*pFUS1-dPSTR*$^{R2-1}$<br>*pAGA1-dPSTR*$^{Y4-3}$	255	255

**Fig. 2C**	yDA296	*pFUS2-dPSTR*$^{R2-1}$<br>*pAGA1-dPSTR*$^{Y4-3}$	267	267
**Fig. 2C**	yDA306	*pPRM1-dPSTR*$^{R2-1}$<br>*pAGA1-dPSTR*$^{Y4-3}$	289	289
**Fig. 2C**	yDA247	*pKAR3-dPSTR*$^{R2-1}$<br>*pAGA1-dPSTR*$^{Y4-3}$	265	265
**Fig. 2C**	ySP643	*pFIG1-dPSTR*$^{R2-1}$<br>*pAGA1-dPSTR*$^{Y4-3}$	224	224
**Fig. 2D**	yDA189	*pFUS1-dPSTR*$^{R2-1}$<br>*pAGA1-dPSTR*$^{Y4-3}$	255	255
**Fig. 2E**	yDA207	*pFIG1-dPSTR*$^{R2-1}$<br>*pKAR3-dPSTR*$^{Y4-3}$	265	265
**Fig. 2F**	yDA207	*pFIG1-dPSTR*$^{R2-1}$<br>*pKAR3-dPSTR*$^{Y4-3}$	265	265
**Fig. 2F**	ySP642	*pAGA1-dPSTR*$^{R2-1}$<br>*pAGA1-dPSTR*$^{Y4-3}$	283	283
**Fig. 2F**	yDA189	*pFUS1-dPSTR*$^{R2-1}$<br>*pAGA1-dPSTR*$^{Y4-3}$	255	255
**Fig. 2F**	ySP643	*pFIG1-dPSTR*$^{R2-1}$<br>*pAGA1-dPSTR*$^{Y4-3}$	224	224
**Fig. 3E**	ySP643	*pFIG1-dPSTR*$^{R2-1}$<br>*pAGA1-dPSTR*$^{Y4-3}$	224	173
**Fig. 3E**	yDA160	*pFIG1-dPSTR*$^{R2-1}$<br>*pAGA1-dPSTR*$^{Y4-3}$<br>*kar4Δ*	232	100
**Fig. 3E**	ySP642	*pAGA1-dPSTR*$^{R2-1}$<br>*pAGA1-dPSTR*$^{Y4-3}$	283	281
**Fig. 3E**	yDA348	*pAGA1-dPSTR*$^{R2-1}$<br>*pAGA1-dPSTR*$^{Y4-3}$<br>*kar4Δ*	320	315
**Fig. 3E**	yDA228	*pFIG1*AGA1-dPSTR*$^{R2-1}$<br>*pAGA1-dPSTR*$^{Y4-3}$	350	305
**Fig. 3E**	yDA234	*pFIG1*AGA1-dPSTR*$^{R2-1}$<br>*pAGA1-dPSTR*$^{Y4-3}$<br>*kar4Δ*	186	109
**Fig. 3E**	yDA313	*pFIG1syn_PREII*^{nc}*_to_consens*<br>*us-dPSTR*$^{R2-1}$<br>*pFIG1-dPSTR*$^{Y4-3}$	287	258
**Fig. 3E**	yDA326	*pFIG1syn_PREII*^{nc}*_to_consens*<br>*us-dPSTR*$^{R2-1}$<br>*pFIG1-dPSTR*$^{Y4-3}$<br>*kar4Δ*	225	169
**Fig. 3E**	yDA352	*pFIG1syn_PREII*^{nc}*_to_consens*<br>*us*AGA1 -dPSTR*$^{R2-1}$<br>*pFIG1-dPSTR*$^{Y4-3}$	165	159
**Fig. 3E**	yDA358	*pFIG1syn_PREII*^{nc}*_to_consens*<br>*us*AGA1-dPSTR*$^{R2-1}$<br>*pFIG1-dPSTR*$^{Y4-3}$<br>*kar4Δ*	357	329
**Fig. 4B**	ySP643	*pFIG1-dPSTR*$^{R2-1}$<br>*pAGA1-dPSTR*$^{Y4-3}$	3500	455*
**Fig. 4C**	ySP643	*pFIG1-dPSTR*$^{R2-1}$<br>*pAGA1-dPSTR*$^{Y4-3}$	3500	455*
**Fig. 4D**	yDA198	*pKAR4-dPSTR*$^{R2-1}$<br>*pAGA1-dPSTR*$^{Y4-3}$	1229	86*

**Fig. 4D**	yDA189	*pFUS1-dPSTR*$^{R2-1}$<br>*pAGA1-dPSTR*$^{Y4-3}$	3283	394*
**Fig. 4D**	yDA302	*pFIG2-dPSTR*$^{R2-1}$<br>*pAGA1-dPSTR*$^{Y4-3}$	3033	455*
**Fig. 4D**	yDA249	*pPRM3-dPSTR*$^{R2-1}$<br>*pAGA1-dPSTR*$^{Y4-3}$	1800	90*
**Fig. 4E**	ySP643	*pFIG1-dPSTR*$^{R2-1}$<br>*pAGA1-dPSTR*$^{Y4-3}$	3500	455*
**Fig. 4E**	yDA198	*pKAR4-dPSTR*$^{R2-1}$<br>*pAGA1-dPSTR*$^{Y4-3}$	1229	86*
**Fig. 4E**	yDA189	*pFUS1-dPSTR*$^{R2-1}$<br>*pAGA1-dPSTR*$^{Y4-3}$	3283	394*
**Fig. 4E**	yDA302	*pFIG2-dPSTR*$^{R2-1}$<br>*pAGA1-dPSTR*$^{Y4-3}$	3033	455*
**Fig. 4E**	yDA249	*pPRM3-dPSTR*$^{R2-1}$<br>*pAGA1-dPSTR*$^{Y4-3}$	1800	90*
**Fig. 4E**	yDA306	*pPRM1-dPSTR*$^{R2-1}$<br>*pAGA1-dPSTR*$^{Y4-3}$	1992	259*
**Fig. 4E**	yDA296	*pFUS2-dPSTR*$^{R2-1}$<br>*pAGA1-dPSTR*$^{Y4-3}$	3200	288*
**Fig. 4E**	yDA247	*pKAR3-dPSTR*$^{R2-1}$<br>*pAGA1-dPSTR*$^{Y4-3}$	3383	203*
**Fig. 4E**	ySP642	*pAGA1-dPSTR*$^{R2-1}$<br>*pAGA1-dPSTR*$^{Y4-3}$	2800	224*

*) Number of fusion events

Appendix Table S4: Oligonucleotides for MNase nucleosome mapping.

Oligos for *AGA1* gene				
**Amplicon**	**Forward_primer**	**Position from ATG**	**Reverse_primer**	**Position from ATG**
**-928**	CACTTCCAAGCGTATCATCAGTT	-977	CAACACAGCATTTGGACCTG	-878
**-858**	ACGTTTGATGCAGGTCCAA	-907	ATCCAGGAACAGAGCCAAACA	-808
**-768**	TCTGTTCCTGGATGGGACAA	-820	GCAACTCAAGATCCAATTCACG	-715
**-679**	TGGATCTTGAGTTGCAAAAGG	-729	GACCAGTCTTTGCGTCAATCA	-628
**-570**	TGGTCTACCAAAGGAATAAGATCAA	-632	TAAATTGAAGCTTGGTCGTTCC	-507
**-488**	CGGTATTGGTCGGAACGA	-539	AAAAGCAAAAGAGTAGGCATCAAA	-437
**-405**	GATGCCTACTCTTTTGCTTTTTCA	-457	GGGTGGACGTACTTGTGCTAA	-353
**-342**	CGTAGCTGTCTAACAGCACCACT	-395	GAAGATATGTGACAGGTACCCTAAATG	-289
**-259**	AGGGTACCTGTCACATATCTTCTCA	-310	ATTATGTTACAGCCGCGTTTTG	-207
**-182**	CAAGTCAAAACGCGGCTGT	-233	TTGCTTGCTGGGAACTGC	-131
**-92**	GCAGTTCCCAGCAAGCAA	-148	AATGCTCGCCGTTTTTCA	-35
**10**	AAAACGGCGAGCATTAACAA	-49	CAAGGCAATATTAGTTAATCCCAACA	69
**102**	TGGGATTAACTAATATTGCCTTGG	47	TAGCGCGGGTGAAACTGTAG	156
**195**	TCACCCGCGCTAGTCTCC	145	CGGCAGCAGATGAAGTGG	244
**268**	ACTTGGTGTCCATTGACGGTA	205	TGCCTCATGGGAGCAGAC	330
**374**	TGCTCCCATGAGGCATGT	316	AATGAGCTGATAGCGGTTGTG	431
**470**	CGCTATCAGCTCATTATCCGAAGT	417	CGAAAGTGTAGAGGTGACAGGTG	522
**Oligos for *FIG1* gene**				
**Amplicon**	**Forward_primer**	**position from ATG**	**Reverse_primer**	**position from ATG**
**-933**	TGATCAACCAAACGCCGATA	-995	CTTGGAAAGTTGGGGCATC	-871
**-833**	TGCCCCAACTTTCCAAGA	-887	GGGAAGACACTGGGTCATTG	-778
**-738**	CAATGACCCAGTGTCTTCCCTA	-797	GAACGTTTGCGTCCGTGTC	-679
**-629**	ATCGACACGGACGCAA	-700	TCACCGGCATTCTTGGAA	-558

	AC			
**-517**	TTCCAAGAATGCCGGTGA	-575	TCATCCCAAAGAGGAAGCAC	-458
**-428**	GTGCTTCCTCTTTGGGATGA	-477	TCGTCTCATCAAGTCAAAATTCG	-378
**-337**	GTGCTTCCTCTTTGGGATGA	-477	TAACTGACACATACATGAAACCATC	-197
**-211**	TGAAAGTCCTTCTCGCTTTAGG	-264	TTTCTTGGTTCGTTTCATTGC	-157
**-123**	AAATATGGCTAAGTAGCAATGAAACG	-192	ACCAAAGACAAGCAAGAACCTG	-54
**-14**	CAGGTTCTTGCTTGTCTTTGGT	-75	TCTGGGCATACGCTTGGTA	48
**61**	TGGTCGCAATCTCAATGATTT	2	CGGGTTGTAACAGCCGATG	120
**168**	GGCTGTTACAACCCGTCAAA	106	TCCTCCAAGCCCAGAGTTG	230
**277**	TGGGCTTGGAGGAAGTCA	218	GCATAGCAAATTGAATTGGAGAA	335
**380**	TTGCTATGCAAGAAAGAATTTAAGC	327	GACAACGCTTGATTGGGTTTT	432
**463**	AAACCCAATCAAGCGTTGTCT	413	TGTTAGGATTACAGTTGCCATCAA	513
**591**	TGTAACTGTCCCTAAGTTACCATTCAA	540	GTCCACATCGCACCAATACC	641
**672**	TGGGGTATTGGTGCGATG	619	GCTGCCTTCTTGCCCTTC	725
**778**	GGGCAAGAAGGCAGCAGT	711	TTTTGGGCACATGGAACATT	845
**887**	AATGTTCCATGTGCCCAAAA	826	TGACATTCTGAAATATCCGCTCA	948
