# Supplementary material for: Timing of gene expression in a cell‐fate decision system
Source: Mol Syst Biol. 2018 Apr 25;14(4):e8024. doi: 10.15252/msb.20178024 (PMC5916086; doi:10.15252/msb.20178024)
Supplement: Supplementary file 2 — Expanded View Figures PDF [file MSB-14-e8024-s002.pdf]

Expanded View Figures

Figure EV1. Dynamics and expression level of mating-dependent promoters.

A–N Population median (solid line) of the nuclear enrichment of the red $dPSTR^R$ (left axis) or the p*AGA1*-$dPSTR^Y$ (right axis, yellow curves) for the 14 promoters of the study. Panels (A–F): early promoters, panels (G–K): intermediate promoters, panels (L–N): late promoters. Note that the scale of the $dPSTR^Y$ is identical for all graphs, whereas $dPSTR^R$ scales are different. The basal level and induced level can vary according to the measured promoter. For instance, p*FAR1* has a high basal level, due to its cell cycle-dependent induction.

O–Q Similar graphs for strains carrying different combinations of dPSTRs.

Data information: In all graphs, the solid line is the median response and the shaded areas represent the 25th–75th percentiles of the single-cell responses. The curves are one representative experiment from at least three replicates.

 © 2018 The Authors

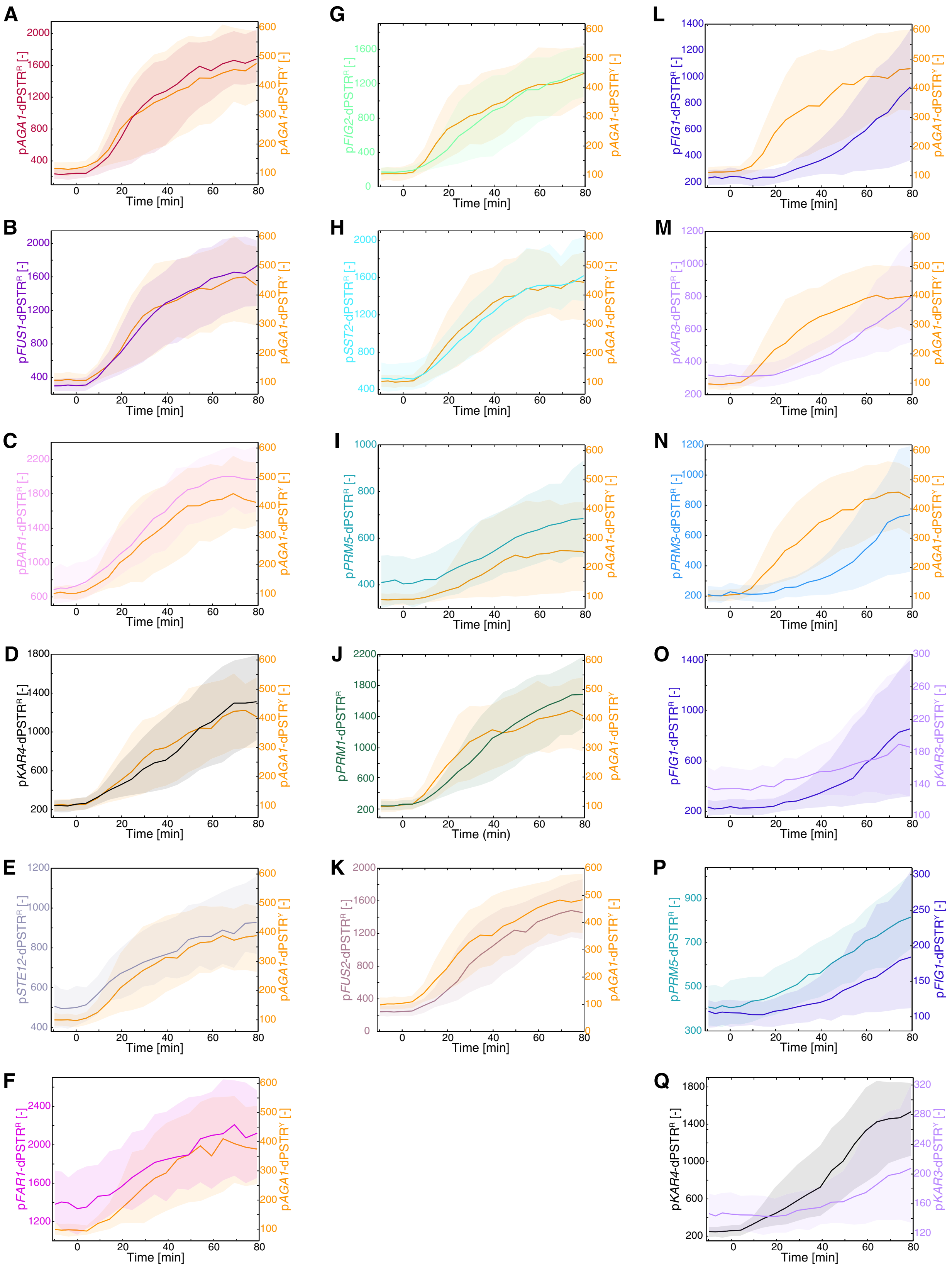

Figure EV1.

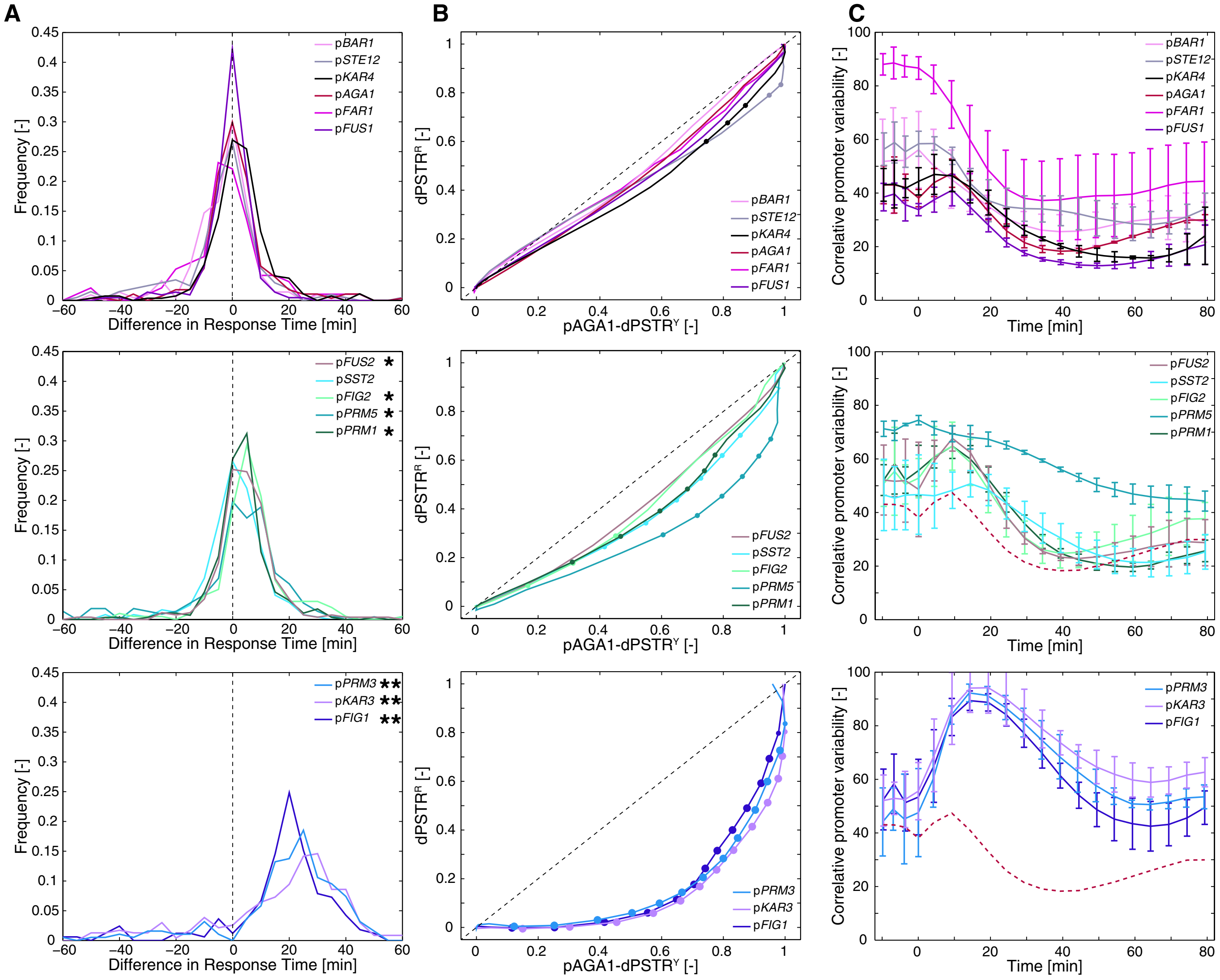

Figure EV2. Characterization of promoters relative to p*AGA1*.

A Distribution of the difference between the response time for p*AGA1*-dPSTR^Y and the specified promoter measured with dPSTR^R for one representative experiment. $N_C > 100$ cells (see Materials and Methods). A sign test was performed to assess distribution centered around 0 (*$10^{-20} < P < 10^{-5}$; **$P < 10^{-20}$).

B Correlation of the average normalized nuclear enrichment of p*AGA1*-dPSTR^Y and specified promoters measured with dPSTR^R at all time points of the experiments. The dotted line is the $x = y$ line and indicates the time direction (from bottom left to upper right). Each curve starts at 0 at the beginning of the experiment. The dots represent the *P*-value ($10^{-3} < P < 10^{-6}$ for small dots and $P < 10^{-6}$ for big dots) of the *t*-test comparing the offset of the measured promoter from the $x = y$ line to the offset of the reference promoter p*AGA1* (red curve, upper panel).

C Quantification of the correlative promoter variability between the indicated promoter measured by dPSTR^R and the p*AGA1*-dPSTRY (see Materials and Methods). Each curve represents the average of the CPV calculated for at least three biological replicates with the standard deviation of the three experiments. The dotted line is p*AGA1*-dPSTR^R curve for comparison.

 © 2018 The Authors

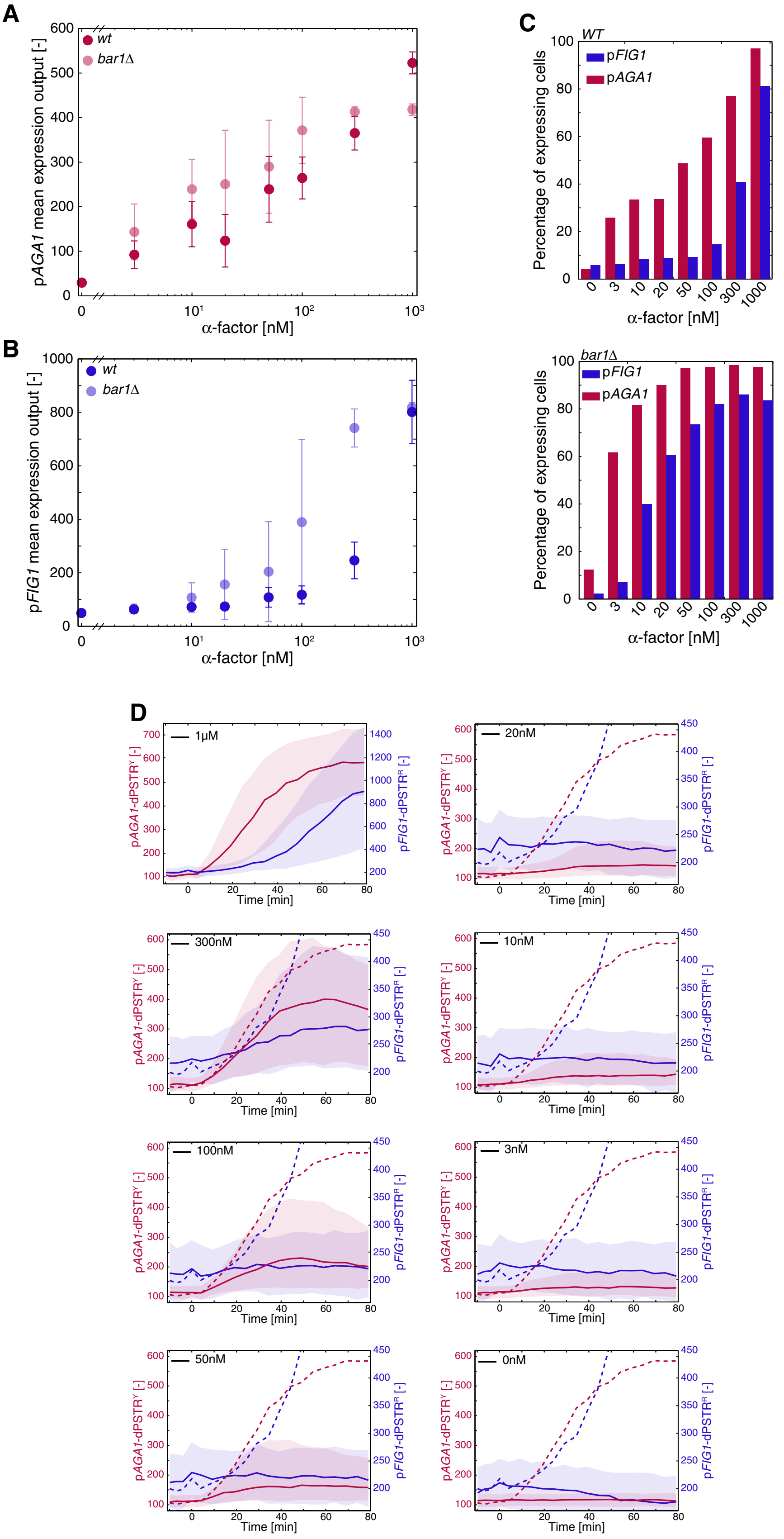

Figure EV3. Dose response of pAGA1 and pFIG1 induction.

A, B Mean expression output for p*AGA1* (A) and p*FIG1* (B) in response to different pheromone concentrations, in a WT or *bar1*Δ (shaded) background. The expression output is defined as the maximal dPSTR nuclear enrichment following stimulation, for all cells of the experiment. Error bars represent the standard deviation of three replicates. Note that the induction of p*AGA1* gradually increases with the pheromone concentration, whereas p*FIG1* displays a switch-like response in WT. Note that in a *bar1*Δ background, the expression occurs at lower concentrations and with higher level for both promoters. The first dot is the non-induced control.

C Percentage of cells expressing p*AGA1* (red) or p*FIG1* (blue) in a WT (upper panel) or *bar1*Δ (lower panel) background, at various pheromone concentrations for one representative experiment. Note that only at high concentrations, a significant proportion of the population expresses p*FIG1*.

D Median nuclear enrichment of the p*AGA1*-dPSTR^Y (red, left axis) and of the p*FIG1*-dPSTR^R (blue, right axis) in course of time, for the different pheromone concentration, in the WT background, for one representative experiment. The solid line is the median, and the shaded area represents the 25th–75th percentile. The reference curves at 1 μM are represented in dashed line for comparison. Note that the scale of the p*FIG1*-dPSTR^R is different between the 1 μM and the other concentrations.

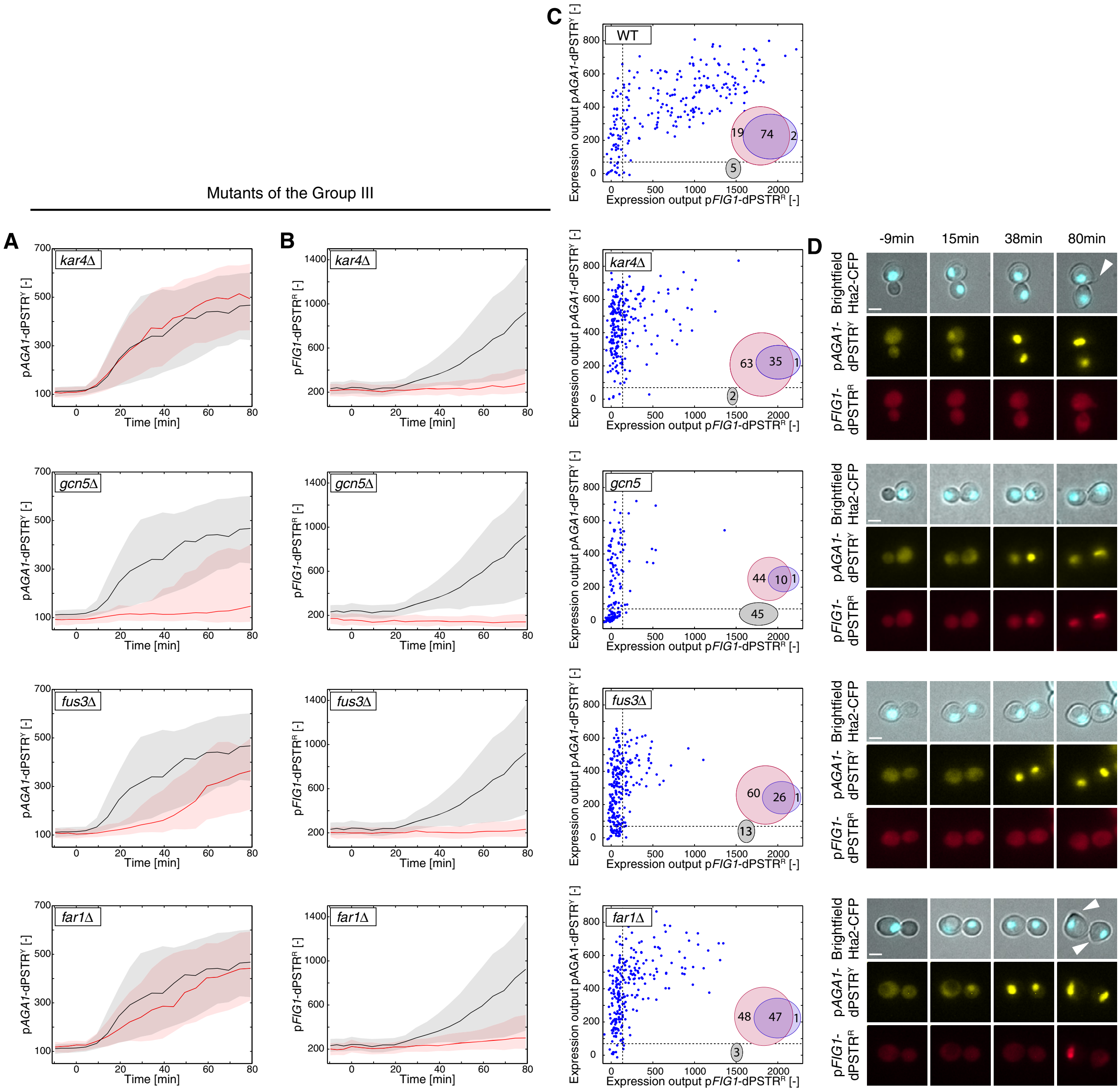

Figure EV4. Mutants of the Group III have differentially impaired induction of pAGA1 and pFIG1.

A, B Nuclear enrichment of the p*AGA1*-dPSTR^Y (A) and p*FIG1*-dPSTR^R (B) after stimulation by 1 μM of pheromone in the indicated mutant. Lines represent the median of either the mutant (red) or the WT strain (black) for one representative experiment, with the solid line representing the median and the shaded area representing the 25th–75th percentile.

C Correlation of the expression output (maximal dPSTR nuclear enrichment following stimulation) of p*AGA1*-dPSTR^Y and p*FIG1*-dPSTR^R for all single cells of the experiment, for the indicated strain. Dotted lines represent the threshold of expression (defined as the 20% of the WT mean expression output for each dPSTR). The Venn diagram represents the proportion of cells expressing p*AGA1* (red circle) or p*FIG1* (blue circle) or none of them (black circle).

D Representative microscopy images of the indicated mutant at the specified time point of the experiment. Arrows indicate shmooing events. Scale bars represent 2.5 μm.

 © 2018 The Authors

Figure EV5. Effect of the loss of Kar4 on the induction of various promoters.

A Nuclear enrichment of the $dPSTR^R$ measuring the indicated promoter in a WT (same color as the axis) or a *kar4*Δ background (red). The solid line is the median, and the shaded area represents the 25th–75th percentiles of the population.

B Distribution of the response time of each promoter in a WT (same color as in A) or a *kar4*Δ background (red).

C Correlation of the expression output (maximal dPSTR nuclear enrichment following stimulation) of the promoters $dPSTR^R$ with the p*AGA1*-$dPSTR^Y$ in a WT (same color as in A and B) or in a *kar4*Δ background (red) for all single cells of the experiment.

© 2018 The Authors

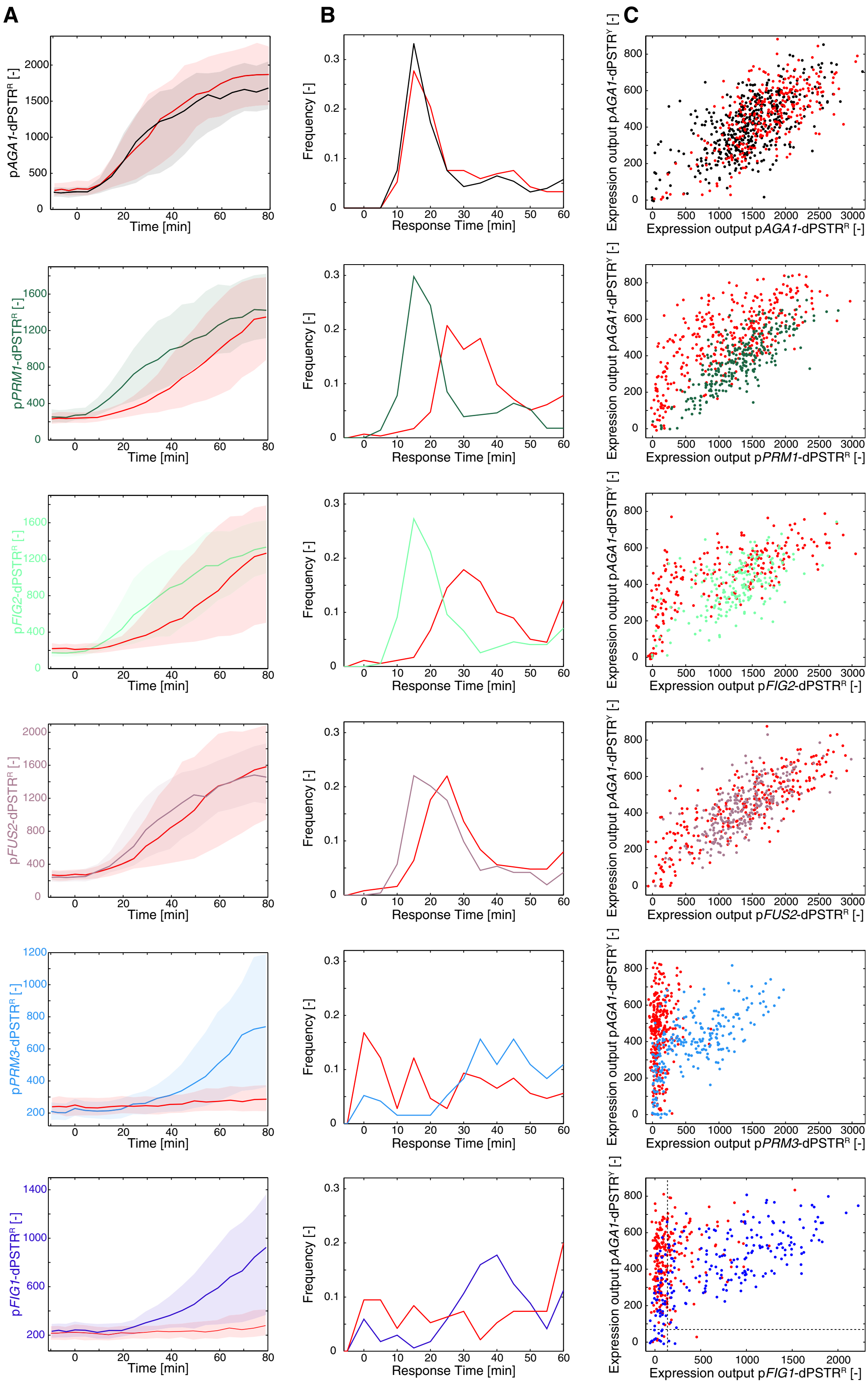

Figure EV5.

 © 2018 The Authors
